# Eco-Friendly Crop Protection: *Argyrantemum frutescens*, a Source of Biofungicides

**DOI:** 10.3390/plants14070985

**Published:** 2025-03-21

**Authors:** Eduardo Hernández-Álvarez, Samuel Rodríguez-Sabina, Noelia Labrador-García, Javier Hernández Pérez, Carolina P. Reyes, María Ángeles Llaría-López, Ignacio A. Jiménez, Isabel L. Bazzocchi

**Affiliations:** 1 Instituto Universitario de Bio-Orgánica Antonio González, Departamento de Química Orgánica, Universidad de La Laguna, Avenida Astrofísico Francisco Sánchez 2, 38206 La Laguna, Tenerife, Spain; alu0100947311@ull.edu.es (E.H.-Á.); alu0101028057@ull.edu.es (N.L.-G.); alu0101230672@ull.edu.es (J.H.P.); ignadiaz@ull.edu.es (I.A.J.); 2 Departamento de Botánica, Ecología y Fisiología Vegetal, Universidad de La Laguna, Avenida Astrofísico Francisco Sánchez, 38206 La Laguna, Tenerife, Spain; srodrisa@ull.edu.es; 3 Instituto Universitario de Bio-Orgánica Antonio González, Departamento de Bioquímica, Microbiología, Biología Celular y Genética, Universidad de La Laguna, Avenida Astrofísico Francisco Sánchez 2, 38206 La Laguna, Tenerife, Spain; cpreyes@ull.edu.es; 4 Área de Gestión del Medio Natural y Seguridad, Cabildo Insular de Tenerife, C/Las Macetas s/n, Pabellón Insular Santiago Martín, 38108 La Laguna, Tenerife, Spain; mllaria@tenerife.es

**Keywords:** *Argyranthemum frutescens*, cultivation, phytopathogens, biofungicides, polyacetylenes

## Abstract

Plant-derived biopesticides are emerging as a promising and popular alternative for promoting cleaner and safer agricultural practices. The present work aims to explore *Argyranthemum frutescens* (*Asteraceae*) as a source of botanical pesticides and to validate this through a cultivation process. To this task, a bioassay-guided fractionation of the ethanolic root extracts from both wild and cultivated *A. frutescens* on phytopathogenic fungi of *Botrytis cinerea*, *Fusarium oxysporum*, and *Alternaria alternata* was conducted. This approach led to the identification of polyacetylenes with higher potency than commercial fungicides. Specifically, compounds **3** (capillin) and **5** (frutescinone) showed more than 90% growth inhibition at 0.05 mg/mL concentration on *B. cinerea*, while compounds **2** (capillinol) and **3** were also more active than positive controls, Fosbel-Plus and Azoxystrobin, against *F. oxysporum*. The structures of the isolated polyacetylenes (**1**–**6**, **9**, and **10**) and alkamides (**7**, **8**, and **11**) were determined through spectroscopic analysis, and the absolute configuration of stereocenter C1 of compounds **1**, **2**, **4** and **9** was determined by NMR-spectroscopy with (*R*)-(-)-α-methoxy-phenylacetic as a chiral derivatizing agent, and biogenetic considerations. Overall, this study supports the potential of polyacetylenes as promising agrochemical lead compounds against phytopathogens, and validates *A. frutescens* cultivation as a viable source of biopesticides.

## 1. Introduction

Plant diseases result in a variety of direct and quantifiable economic consequences for crop management [1]. In particular, phytopathogenic fungi represents a significant threat to global food security, ecosystems, and human health [2]. Among the phytopathogenic fungi, *Botrytis cinerea*, *Fusarium oxysporum* and *Alternaria alternata*, are considered high-risk plant pathogens affecting crops worldwide [3]. In fact, *A. alternata* is one of the most common and cosmopolitan species causing disease on economically important crops due to its environmental adaptability to UV light, low temperature, and water stress conditions [4]. Dark sunken lesions are usually the expression of *Alternaria* spp. infections on roots, tubers, stems, and fruits in a wide range of vegetable- and fruits-producing plants. The *Alternaria* toxins as natural contaminants in food are a serious risk to human and animal health because of their known toxicity, since their administration/ingestion has been shown to be cytotoxic, fetotoxic, and teratogenic in animal; mutagenic in microbial and mammalian cell systems; and tumorigenic in rats [5]. *B. cinerea* is a widespread plant pathogen with a necrotrophic lifestyle, causing overwhelming diseases in more than 1400 plant species, especially fruit crops, resulting in significant economic losses worldwide [6]. The pathogen causes rotting of fruits at both preharvest and postharvest stages, and aside from causing “gray mold” on the mature fruits, the fungus infects leaves, flowers, and seeds. *B. cinerea* produces two major phytotoxins involved in the infection caused by this fungus, the sesquiterpene botrydial and the polyketide botcinin acid [7]. Many *Fusarium* species, such as *F. oxysporum,* are pathogenic, causing crop diseases during crop production and spoilage of agricultural products in both commercial and smallholder farms. The damage caused by this fungus is through their direct attack of crops in the fields and by the production of allergenic compounds and mycotoxins, causing yield loss and increases in food insecurity and food prices [8].

To minimize the crop losses, synthetic fungicides are widely used, despite their overapplication or misuse having raised serious concerns, including their impact on the environment, and the effect on human health and livestock [9]. These harmful effects, combined with the induction of resistance development in pests [10], have promoted the introduction of sustainable, effective, and environmentally friendly alternatives to the use of chemicals in crops protection [11]. In this regard, biopesticides have demonstrated the potential to be used to protect crops against phytopathogens, reducing the use of conventional synthetic fungicides as a vital step towards sustainable crop production [12]. Plant-derived pesticides have great advantages, being effective against various pests, biodegradable, abundant, and less expensive [13].

Moreover, the discovery of biopesticides from a plant species could lead to the overexploitation of natural habitats [14], and therefore, there is a need to find sustainable sources of biomass to prevent an ecological crisis. In this regard, the cultivation of a target species as a source of biopesticides arises as a sustainable strategy to ensure the availability of biomass [15].

The *Asteraceae* family is the most extensive within the angiosperms, and it is distributed worldwide [16]. Species of this botanical family have extensive pharmaceutical applications, including antioxidant, anti-inflammatory, and antibacterial [17], but also pesticide properties [13,18]. Recent studies reported that plants belonging to this family have an excellent ability to synthesize nanoparticles in non-toxic ways having numerous applications [19]. The phytochemical profile of plants in the *Asteraceae* family reveals a multitude of bioactive compounds, such as terpenes, saponins, lignans, and polyphenolic compounds, which are responsible for their wide range of properties [13,20]. In particular, the genus *Argyranthemum* Webb ex Sch.Bip., the largest genus of flowering plants endemic to the Macaronesian archipelagos, is present in all the major habitat zones in Macaronesia, ranging from coastal to subalpine habitats, and comprises twenty-four species [21]; three of them are endemic to Madeira, one to Selvagens and twenty to the Canary Islands. *Argyranthemum frutescens* (L.) Sch. Bip. subsp. *frutescens* named as “magarza” is a perennial plant originating in the Canary (Spain) and Madeira (Portugal) Islands [22] used in stomach and buccal tonics and for asthma treatment [23]. Moreover, its potential as a biopesticide has not been investigated.

The present research provides a comprehensive account of the isolation and structural elucidation of eight polyacetylenes and three alkamides from the ethanolic extracts of the roots of *A. frutescens*, wild as well as cultivated, through a bioassay-guided fractionation targeting three strains of phytopathogenic fungi, *A. alternata*, *B. cinerea* and *F. oxysporum*. Evaluation of the isolated metabolites pointed out the polyacetylenes as promising components to be used as biopesticides. The preliminary structure–activity relationship is also discussed.

## 2. Results and Discussion

### 2.1. Bioassay-Guided Fractionation

In recent decades, the use of bioassay-guided fractionation techniques have been rising as the main tool to decipher in the discovery and development of bioactive compounds from natural resources. They are used in the early stages of compounds isolation/purification/identification, serving as a starting point for application-based development [24].

**Figure 1:**
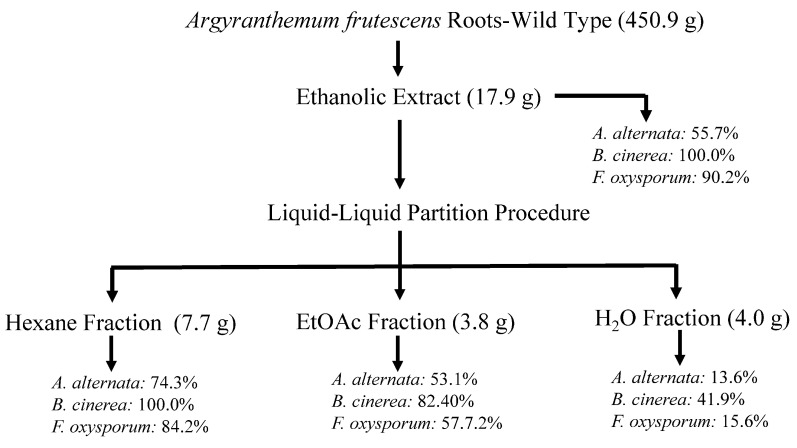
Flowchart of bioassay-guided fractionation of wild *A. frutescens* roots on the mycelium stage of *Alternaria alternata*, *Fusarium oxysporum,* and *Botrytis cinerea*. Values represent the percentage of growth inhibition (%GI) at 1 mg/mL concentration. Fosbel-Plus (%GI 93.5, 83.3, and 93.4, respectively) was used as a positive control.

Previous studies on *A. frutescens* conducted three decades ago reported the isolation of acetylenes and an alkamide from various parts of the plant [25], as well as the antimicrobial and cytotoxic activities of the acetone extract from the roots and its isolated metabolites [26]. In this study, as part of our research program aimed at discovering plant-derived biopesticides, we carried out a bioassay-guided fractionation of the ethanolic extracts from both wild-type and cultivated *A. frutescens* roots against three phytopathogenic fungi affecting crops worldwide (Figure 1, and Table S1 in Supporting Materials). Growth inhibition percent (%GI) was determined by a test on the mycelium stage [27] of the phytopathogens: *Botrytis cinerea*, *Fusarium oxysporum*, and *Alternaria alternata*. Samples exhibiting %GI higher than 20% at 1 mg/mL were also evaluated at 0.5 and 0.1 mg/mL doses. Commercial antifungal agents, Fosbel-Plus (35% Fosetil Al and 35% Mancozeb) and Ortiva PC (Azoxystrobin 250 g/L, 22.8% p/p) in the case of *B. cinerea* (since Fosbel-Plus was inactive at the lower concentrations assayed), were used as positive controls for comparative purposes in the current analysis, whereas ethanol was used as a negative control.

Thus, the air-dried and powdered roots of wild-type *A. frutescens* plant (450.9 g) were extracted by maceration with 96% EtOH (5 L, 24 h, three times) at room temperature (22 ± 4 °C). This procedure yielded 17.9 g of residue after the solvent was removed under reduced pressure at 40 °C (4.0% *w*/*w*), and an aliquot of this extract was assayed on phytopathogenic fungi under study. The EtOH extract from the wild-type plant showed remarkable activity at 1 mg/mL concentration with an inhibition growth (%GI) of 55.7%, 100.0% and 90.2% on *A. alternata*, *B. cinerea* and *F. oxysporum*, respectively, showing some degree of activity on *B. cinerea* (47.0%) even at 0.1 mg/mL (Figure 1 and Table S1 in Supporting Materials). These promising results encouraged us to achieve a bioassay-guided fractionation. Therefore, the EtOH extract was successively partitioned into hexane (Hx), EtOAc, and H_2_O fractions by liquid–liquid partition, and the fractions were further evaluated. The results revealed that the two organic fractions exhibited activity against the tested fungi, highlighting the Hx fraction with GI values ranging from 74.3 to 100.0% at 1 mg/mL, more potent than the commercial antifungal Fosbel-Plus on *B. cinerea* (GI 100 vs. 83.3%) and slightly lower against *F. oxysporum* (GI 84.2 vs. 93.4%) at 1 mg/mL concentration. By contrast, the aqueous fraction was inactive (%GI < 10 at 1 mg/mL) against two of the phytopathogens, with moderate effect on *B. cinerea* (GI 41.9% at 1 mg/mL).

These promising results regarding the activity of the wild-type *A. frutescens* roots on the assayed phytopathogenic fungus encouraged us to search for a sustainable source of this plant species. In this regard, cultivation of a species would not only provide the possibility of managing the production and preservation of the species but also lead to the production of uniform raw materials, and the possibility of production in all seasons according to the climatic diversity of the country [15].

**Figure 2:**
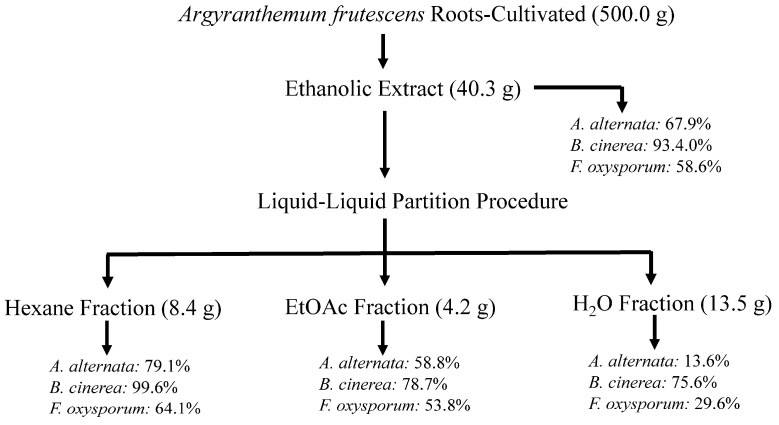
Flowchart of bioassay-guided fractionation of cultivated *A. frutescens* on the mycelium stage of *Alternaria alternata*, *Fusarium oxysporum*, and *Botrytis cinerea*. Values represent the percentage of growth inhibition (%GI) at 1 mg/mL concentration. Fosbel-Plus (GI 93.5, 83.3, and 93.4%, respectively) was used as a positive control.

Therefore, the species *A. frutescens* was cultivated as described in detail in the Experimental section. The air-dried and powdered roots (500.0 g) were extracted by maceration with 96% EtOH at room temperature (22 ± 4 °C), yielding 40.3 g of residue (8.1% *w*/*w*) after the solvent was removed. Following the same procedure as that for the wild-type plant, this EtOH extract was evaluated on the three phytopathogenic fungi, and results indicated some degree of activity against the assayed fungi (67.9% on *A. alternata,* 93.4% on *F. oxysporum,* and 58.6% on *B. cinerea* at 1 mg/mL concentration), although it was lower than the EtOH extract from the wild-type plant on *F. oxysporum* (Figure 2). 

This ethanolic extract was subjected to fractionation through a liquid–liquid partition and then to biological evaluation, revealing that the hexane fraction showed the highest efficacy with GI of 79.1, 99.6 and 64.1% at 1 mg/mL concentration against *A. alternata*, *B. cinerea,* and *F. oxysporum,* respectively, similar to the extract from the wild-type plant (Figure 2).

**Figure 3:**
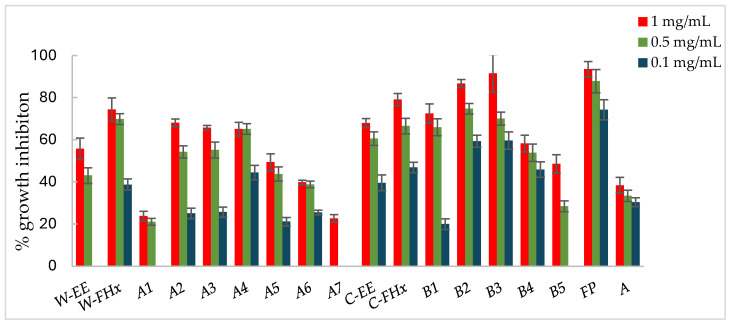
Antifungal effects (% growth inhibition, %GI) of *A. frutescens* roots against *Alternaria alternata*. Wild-type and cultivated plants: ethanolic extracts (W-EE and C-EE), hexane fractions (W-FHx and C-FHx), and subfractions (A1–A7 and B1–B5). Subfractions with a GI higher than 20% at 1 mg/mL were assayed at lower concentrations (0.5 and 0.1 mg/mL). Subfractions with GI < 20% have been omitted. Results are expressed as percentage relative to the negative control. Fosbel-Plus and Azoxystrobin were used as positive controls. Data are presented as mean ± SD (standard deviation, *n* = 8); *p* < 0.05.

**Figure 4:**
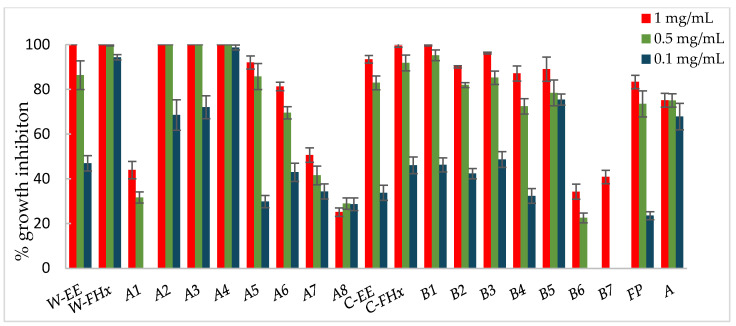
Antifungal effects (% growth inhibition, %GI) of *A. frutescens* roots against *Botrytis cinerea*. Wild-type and cultivated plants: ethanolic extracts (W-EE and C-EE), hexane fractions (W-FHx and C-FHx), and subfractions (A1–A8 and B1–B7). Subfractions with a GI higher than 20% at 1 mg/mL were assayed at lower concentrations (0.5 and 0.1 mg/mL). Subfractions with GI < 20% have been omitted. Results are expressed as percentage relative to the negative control. Fosbel-Plus and Azoxystrobin were used as positive controls. Data are presented as mean ± SD (standard deviation, n = 8); *p* < 0.05.

**Figure 5:**
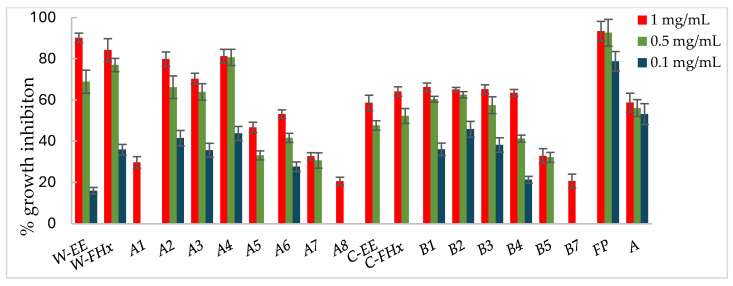
Antifungal effects (% growth inhibition, %GI) of *A. frutescens* roots against *Fusarium oxysporum*. Wild-type and cultivated plants: ethanolic extracts (W-EE and C-EE), hexane fractions (W-FHx and C-FHx), and subfractions (A1–A8 and B1–B7). Subfractions with a GI higher than 20% at 1 mg/mL were assayed at lower concentrations (0.5 and 0.1 mg/mL). Subfractions with GI < 20% have been omitted. Results are expressed as percentage relative to the negative control. Fosbel-Plus and Azoxystrobin were used as positive controls. Data are presented as mean ± SD (standard deviation, n = 8); *p* < 0.05.

Consequently, the hexane fractions emerge as the most promising ones to advance in the bio-guided fractionation process, and those were selected to proceed with the bio-guided fractionation, investigating the metabolites responsible for bioactivity. Thus, the Hex fractions were chromatographed over column chromatography (hexane/EtOAc of increasing polarity, 10:0 to 0:10 as eluent), and subfractions were combined based on their TLC similarity, yielding subfractions A1–A8 and B1–B7 from the wild-type and cultivated plant materials, respectively. Each fraction was assayed against the phytopathogenic fungi, *A. alternata* (Figure 3), *B. cinerea* (Figure 4), and *F. oxysporum* (Figure 5).

Subfractions A2–A4 exhibited percentage inhibition values > 60% at 1 mg/mL against the three fungi (Figure 3, Figure 4 and Figure 5, Table S1 in Supporting Materials). *B. cinerea* was the most sensitive fungus, with subfractions A2–A4 reaching a 100% of growth inhibition at 0.5 mg/mL, significantly higher than when positive controls, Fosbel-Plus (GI 73.5%) and Azoxystrobin (GI 75.0%) were used. Even subfraction A4 showed a GI of 98.7% at 0.1 mg/mL (Figure 4), the lowest assayed concentration. Regarding *F. oxysporum,* subfraction A4 exhibited a GI of 80.7% at 0.5 mg/mL (Figure 5), whereas *A. alternata* was revealed to be the least sensitive fungus to these subfractions, with the most active subfraction being A4 which showed a GI 65.1% at 0.5 mg/mL (Figure 3), slightly lower than the hexane fraction.

Regarding the results of the subfractions from the cultivated plant against the phytopathogenic fungi (Figure 3, Figure 4 and Figure 5, Table S2 in Supporting Materials), subfractions B1–B5 exhibited similar behavior pattern as the subfractions from the wild-type plant. Thus, *B. cinerea* was the most sensitive fungus, with %GI ranging from 87.1 to 99.8 at 1 mg/mL, higher than the two positive controls (Fosbel-Plus, GI 83.3% and Azoxystrobin, 75.1%) at the same concentration. Also, these subfractions had an excellent activity at 0.5 mg/mL on this fungus (GI from 72.4 to 95.2%). Moreover, subfractions B1–B3 exhibited a significant degree of growth inhibition on *A. alternata* (72.4–91.5%) and *F. oxysporum* (65.1–66.3%) at 1 mg/mL. Moreover, subfractions B6 and B7 were inactive (%GI < 10%) and poorly active on *A. alternaria* and *F. oxysporum*, respectively, and moderately active on *B. cinerea* (%GI 34.3 and 40.9, respectively).

The results indicated that the antifungal activity of the EtOH extract from the cultivated plant is progressively enriched in the hexane fraction and its respective subfractions by liquid–liquid partitioning and column chromatography. This points to a selective concentration of bioactive compounds responsible for activity. Moreover, the results of the wild-type plant extract indicated that the subfractions were from slightly to moderately less active than the hexane fraction on *A. alternata* and *F. oxysporum*, whereas on *B. cinerea* the potency remained at 100% from the EtOH extract to subfractions. Therefore, the cultivated plant exhibited an efficient bioassay-guided isolation process, whereas the wild-type plant demonstrated partial efficiency, suggesting that the antifungal activity arises from interactions among multiple compounds involving potential synergistic effects.

### 2.2. Metabolites Identification from Active Subfractions

**Figure 6:**
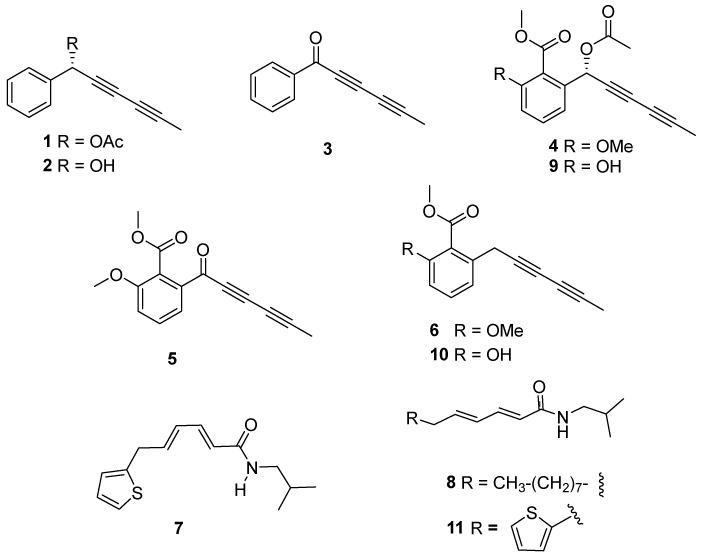
Structure of compounds isolated (**1**–**11**) from wild-type (**1**–**8**) and cultivated (**1**, **2**, **4**, **6**, and **8**–**11**) *Argyranthemum frutescens* roots.

Based on the bioactivity, subfractions A2–A6 and B1–B5 from the wild-type and cultivated plants, respectively, were selected for successive purification steps by column chromatography to identify the components in those bioactive subfractions. This procedure yielded compounds **1**–**11** (Figure 6), the structure elucidation of which was achieved by means of spectroscopic techniques and comparison with previously reported data.

Thus, the isolated compounds were identified as: 1-phenylhexa-2,4-diyn-1-yl acetate (capillinol acetate, **1**) [26], 1-phenylhexa-2,4-diyn-1-ol (capillinol, **2**) [28], capillin (**3**) [28], frutescinol acetate (**4**) [26], frutescinone (**5**) [26], frutescin (**6**) [26], *N*-isobutyl-6-(2-thienyl)-2*Z*,4*Z*-hexadienamide (**7**) [29], 2*E*,4*E*-tetradecadienoic acid isobutyl amide [30] (**8**), 3′-*O*-demethyl frutescinol acetate (**9**) [26], 3′-*O*-demehthyl frutescin [31] (**10**), and *N*-isobutyl-6-(2-thienyl)-2*E*,4*E*-hexadienamide [32] (**11**). Moreover, metabolites **1**–**8** were isolated from the wild-type plant, whereas metabolites **1**, **2**, **4**, **6** and **8**–**11** were identified in the cultivated plant.

**Figure 7:**
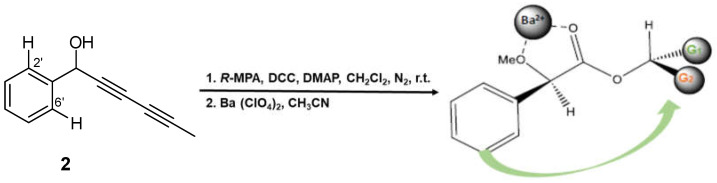
Synthesis of the capillinol (*R*)-α-methoxyphenylacetate and saturation with barium (II) salt as the chelating agent. *R*-MPA: acid, DCC: *N*, *N*′-dicyclohexylcarbodiimide; DMAP: 4-(dimethylamine)pyridine; r.t.: room temperature.

Although the spectroscopic data and relative stereochemistry of compound **2** (capillinol) were previously reported [28], its absolute configuration remained unresolved. Thus, we report the first determination of the absolute configuration of the secondary alcohol on C1 of this compound by Riguera’s method [33], a variation of Mosher’s method, based on the use of (*R*)-(-)-α-methoxyphenylacetic (MPA) as the chiral reagent and barium (II) salt as the chelating agent. This methodology offers the advantage that it requires a small amount of sample; only one of the esters, either (*R*) or (*S*)-MPA, needs to be prepared. The absolute configuration was assigned by comparing the chemical shift of substituent G2 (H-2′ and H-6′) before (δ_H_ 7.4691) and after (δ_H_ 7.3804) saturation with Ba(ClO_4_)_2_ (ΔδBa = +0.0887) in the ^1^H NMR spectra. Applying Riguera’s model (Figure 7), the most shielded substituent aligns facing the phenyl moiety of the chiral ester, thereby dictating the absolute configuration of the secondary alcohol as 1*S*. Moreover, the absolute configuration of this stereocenter in compounds **1**, **4** and **9** was assumed based on biosynthetic considerations, since they contained the same polyacetylene core and specific rotation sign as compound **2**.

### 2.3. Antifungal Activity Assays of the Isolated Compounds

The effects on fungi viability of the eleven compounds (**1**–**11**) isolated from both wild-type and cultivated *A. frutescens* roots were evaluated individually against the three phytopathogenic fungi (Table 1). Compounds with an inhibition growth higher than 20% at 0.1 mg/mL were assayed at lower concentrations (0.05 and 0.01 mg/mL). Fosbel-Plus and Ortiva PC were used as positive controls.

The results showed that compounds **2**, **4** and **9** exhibited good activity against *A. alternata* with %GI values ranging from 56.6% to 71.3%, highlighting compound **9** with slightly lower potency than the positive control, Fosbel-Plus (%GI = 74.2%) at the concentration of 0.1 mg/mL. Moreover, compounds **1**, **5**, **6**, **7,** and **11** exhibited moderate activity against this fungus with GI ranging from 34.6 to 48.1% at 0.1 mg/mL, whereas compounds **3** and **10** showed low potency, and compound **8** was inactive (%GI < 10). Regarding *B. cinerea*, all the assayed compounds, except for compund **8**, showed some degree of antifungal activity. In fact, compounds **1**–**3**, **6**, **9,** and **10** (%GI from 70.1 to 100%), exhibited higher potency than the commercial fungicide, Azoxytrobin (%GI 67.8) at 0.1 mg/mL, highlighting compounds **3** (capillin) and **5** (frutescinone) with %GI 92.7 and 90.8, respectively, at 0.05 mg/mL, and this last one keeping a %GI 70.6 at the lowest assayed concentration. This fungus was the most sensitive one to the assayed compounds, in concordance with the results of the subfractions from the wild-type and cultivated plants. *Fusarium oxysporum* was also sensitive to the compounds and compounds **2** (%GI = 44.5) and **3** (%GI = 84.4) were also more active than the positive controls (Fosbel Plus, %GI = 44.4 and Azoxystrobin, %GI 51.6) at the concentration of 0. 05 mg/mL.

Polyacetylenes are a kind of naturally occurring metabolites broadly present in the *Apiaceae*, *Araliaceae*, and *Asteraceae* families, which have attracted considerable attention owing to their multiple pharmacological effects, including antitumor, anti-inflammatory, antifungal, and antibacterial activities [34,35]. Polyacetylenes are also considered to be phytoalexins, which play an important ecological role in plants’ response to disease states, microbial attacks or abiotic stresses [36]. In fact, some polyacetylenes have been reported to exhibit insecticidal activity and can be used as biological control agents [34], such as atractylodin from *Atractylodes lancea* that shows repellent effects against *Tribolium castaneum*, a worldwide pest of stored products, particularly food grains [37]. Regarding polyacetylenes isolated herein, capillin has been reported to elicit the human leukemia HL-60 cell apoptosis [38] and to inhibit cell proliferation on various human tumor cell lines and the carcinogenic Epstein–Barr virus [39]. In addition, capillin and capillinol hold great promise as anti-diabetic drug candidates [40]. However, to our knowledge, the present work is the first one that reports on their potential as biopesticides.

The results in the present study pointed out not only the potential of *A. frutescens* as a source of polyacetylenes with a potent fungicide activity on high-risk plant pathogens but also validate the cultivated plant species as having similar phytochemical and bioactivity profiles that the wild-type plant.

### 2.4. SAR Analysis

**Figure 8:**
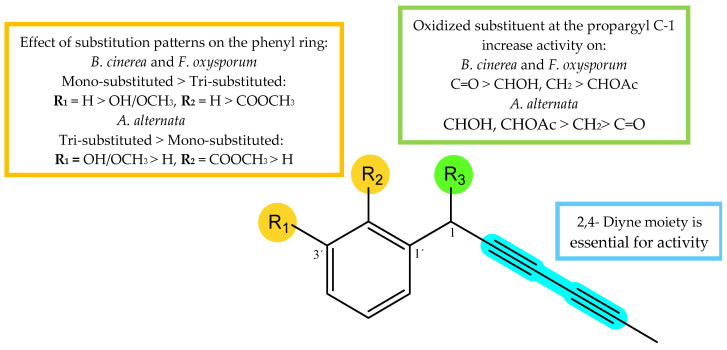
Structure–activity relationship of most active compounds, polyacetylenes (**1**–**6**, **9,** and **10**).

Taking into consideration the obtained %GI values (Table 1) of the assayed compounds on phytopathogenic fungi, the influence of substitution patterns and some structure-activity requirements can be established for the two types of isolated compounds, polyacetylenes (**1**–**6**, **9** and **10**) and alkamides (**7**, **8** and **11**) (Figure 8). (1) Considering the polyacetylene compounds, the results of the analysis revealed the following trends: (a) The type of the functional group at the propargyl C-1 position seems critical for the activity. Thus, the comparison of the activities of polyacetylenes **1**–**3** and **4**–**6** whose only structural difference is the substituent at C-1, showed that the most effective group at this position was the ketone group (**3** and **5**), followed by hydroxyl or methylene (**2** and **6**), and acetyl group (**1** and **4**) for *B. cinerea* and *F. oxysporum*, whereas the results for *A. alternata* showed a different behavior, with a hydroxyl group (**2**) being preferable to a ketone (**3**) or acetyl moiety (**1**). (b) The number of substituents on the aromatic ring modulated the bio-fungicide profile. Thus, compounds with a monosubstituted aromatic ring were more potent than those trisubstituted (**1** vs. **4**, and **3** vs. **5**) on *B. cinerea* and *F. oxysporum*. Furthermore, a carboxy-methyl ester at C-2′ and a methyl ether at C-3′ strongly affect the activity, since their substitution by a hydrogen atom enhances the activity (**5** vs. **3** and **4** vs. **1**). Once again, results for *A. alternata* were contrary, with the trisubstituted polyacetylenes (**4** and **5**) being more active than the monosubstituted ones (**1** and **3**), suggesting that oxidation of the aromatic ring had a favorable effect on the activity against this fungus. (2) Concerning the compounds with an alkamide skeleton, the replacement of a thiophene ring by an alkyl side chain moiety (**8** vs. **11**) significatively decreased the potency.

## 3. Materials and Methods

### 3.1. General

Optical rotations were measured with a Perkin Elmer 241 automatic polarimeter in CHCl_3_ at 20 °C and the [α]_D_ values are given in 10^−1^ deg cm^2^/g. The NMR experiments were recorded using Bruker Avance 500 or 600 spectrometers (Bruker, Wissembourg, France) with the pulse sequences given by Bruker using CDCl_3_ as a solvent, and the chemical shifts are given in δ (ppm) with TMS as internal reference. Silica gel 60 (particle size 15–40 and 63–200 μm, Macherey-Nagel, Düren, Germany) and Sephadex LH-20 (Pharmacia Biotech, Uppsala, Sweden) were used for column chromatography, and silica gel 60 F254 (Macherey-Nagel) was used for analytical or preparative TLC. The spots were visualized by UV light and heating silica gel plates sprayed with H_2_O-H_2_SO_4_-AcOH (1:4:20). All solvents of analytical grade and the reagents were purchased from Panreac (Barcelona, Spain). PGA culture medium (Potato glucose agar, Sigma-Aldrich, Madrid, Spain) and 9 cm diameter Petri dishes (Sarstedt, Nümbrecht, Germany) were used for the maintenance of the fungal colonies, and to carry out bioassays. Tetracycline (50 mg/L, Sigma-Aldrich, Madrid, Spain) was added to avoid bacterial growth on the Petri dishes. The stock solutions of tested samples were prepared with absolute ethanol (Sigma-Aldrich, Madrid, Spain). Fosbel-Plus (35% Fosetil Al and 35% Mancozeb) from Probelte S.A. (Murcia, Spain) was purchased in a phytosanitary product store, and used as a reference fungicide for tests against *A. alternata*, *B. cinerea,* and *F. oxysporum*. Ortiva PC (Azoxystrobin 250 g/L, 22.8% p/p) from Syngenta España S.A (Madrid, Spain) was also used as a reference fungicide. Ethanol was used as a negative control, using one dish per pathogen and eight discs for each control.

### 3.2. Plant Material

#### 3.2.1. Plant Collection

Roots of *Argyranthemum frutescens* (L.) Sch. Bip. subsp. frutescens were gathered at Rambla de Castro, in the Los Realejos municipality, Tenerife Province (Canary Islands, Spain) in May 2023. The botanical identification was performed by botanist Ph D Cristina G. Montelongo, and a voucher specimen (TFC 54141) has been deposited in the Herbarium TFC-SEGAI, Universidad de La Laguna (Tenerife, Spain).

#### 3.2.2. Plant Cultivation: Seedling Production

The seed of the species *A. frutescens* were collected from the natural environment and native plants belonging to the garden center of native flora at the Environmental Centre La Tahonilla (Cabildo Insular de Tenerife, Canary Island, Spain) sited at La Laguna (36°17′25″ N and 59°35′45″ E; 985 m above sea level). The conditions in the greenhouse during the sowing phase were 70–80% humidity and 23 ± 2 °C. This material was cultivated using compost (25%), coconut fiber (50%), and perlite (25%) as substrates and irrigated three times a week. The seedlings with 3–4 leaves were further transplanted into individual pots. In this first phase of growth, plants received only water to maintain optimal conditions, because the application of fungicide can affect the roots’ development. In the potting phase, solid fertilizer (8 gr/each pot, 16N-9P-12K) was added once a month to enhance the blooming. Once they were transplanted into a new pot, they were placed in the experimental field in May 2023 (325 plants) to growth until they were collected. Roots of the cultivated *A. frutescens* were gathered in January 2023. The roots were spread on a tray, turned over occasionally, and air-dried at room temperature (22 ± 4 °C) for two weeks. The dried plant materials were ground and stored until extraction.

### 3.3. Plant Extracts Preparation and Liquid–Liquid Partition Procedure

The air-dried and powdered roots of wild (450.9 g) and cultivated (500.0 g) *A. frutescens* were extracted by maceration with 96% EtOH (5 L, 24 h, three times) at room temperature (22 ± 4 °C). The resulting extracts were filtered, and the solvent removed under reduced pressure at 40 °C. This procedure yielded 17.9 g and 40.3 g of residue (4.0% and 8.1% *w*/*w*) from the wild and cultivated plants, respectively. An aliquot for each one was assayed on phytopathogenic fungi under study, revealing a notable fungal inhibition for both extracts.

Therefore, the ethanolic extracts were subjected to further fractionation through a liquid–liquid partition. Thus, the extracts were suspended in water (500 mL H_2_O) and partitioned sequentially with hexane (Hx, 3 × 500 mL) and ethyl acetate (EtOAc, 3 × 500 mL). The organic phases were concentrated under reduced pressure to give the Hx (7.7 g and 8.4 g for wild and cultivated plants, respectively) and EtOAc (3.8 g and 4.2 g for wild and cultivated plants, respectively) fractions. Simultaneously, the aqueous residues were subjected to lyophilization, yielding the aqueous fractions (4.0 and 13.5 g, respectively). Aliquots (approximately 45 mg) of the fractions from the liquid–liquid protocol were assayed for their antifungal activity. Biological evaluation revealed that the organic fractions were active against the phytopathogenic fungi, with the hexane fraction in both wild and cultivated plants showing the highest efficacy. Therefore, those hexane fractions were further investigated.

### 3.4. Bioactivity-Guided Chromatographic Fractionation and Metabolites Isolation in Wild and Cultivated A. frutescens Roots

The most active fractions from the wild and cultivated *A. frutescens*, the hexane ones (7.7 g and 8.4 g, respectively), were chromatographed on a silica gel column, using a mixture of hexane/EtOAc of increasing polarity (10:0 to 0:10) as eluent to afford subfractions, which were then combined based on their TLC profile into subfractions A1–A8 and B1–B7 (Experimental Part S1 in Supporting Materials). Phytopathogenic fungi evaluation of those fractions revealed that subfractions A2–A6 and B1–B5 were active against the strains of *A. alternata*, *B. cinerea,* and *F. oxysporum*. Therefore, these subfractions were subjected to several chromatography steps until yielding the pure compounds (Figure 1 and Figure 2). Following this procedure, eleven metabolites were isolated and identified by NMR techniques (Figures S1–S10 in Supporting Materials) and compared with those reported in the literature. Thus, compounds identified in the wild plant correspond to: capillinol acetate (**1**, [α]^20^_D_ + 42.2°, *c* 1.68 in CHCl_3_; 80.5 mg) [26], capillinol (**2**, [α]^20^_D_ + 3.0°, *c* 0.44 in CHCl_3_; 19.6 mg) [28], capillin (**3**, 6.4 mg) [28], frutescinol acetate (**4**, [α]^20^_D_ + 8.2°, *c* 0.02 in CHCl_3_; 4.6 mg) [26], frutescinone (**5**, 13.1 mg) [26], frutescin (**6**, 11.4 mg) [26], *N*-isobutyl-6-(2-thienyl)-2*Z*,4*Z*-hexadienamide (**7**, 1.6 mg) [29], and 2*E*,4*E*-tetradecadienoic acid isobutyl amide (**8**, 7.6 mg) [30]. Moreover, the following metabolites were identified in the cultivated plant: capillinol acetate (**1**, 667.4 mg) [26], capillinol (**2**, 69.6 mg) [28], frutescinol acetate (**4**, 78.7 mg) [26], frutescin (**6**, 269.6 mg) [26], 2*E*,4*E*-tetradecadienoic acid isobutyl amide (**8**, 9.4 mg) [30], 3′-*O*-demethyl frutescinol acetate (**9**, [α]^20^_D_ + 14.3°, *c* 0.28 in CHCl_3_; 163.1 mg) [26], 3′-*O*-demehthyl frutescin (**10**, 8.7 mg) [31], and *N*-isobutyl-6-(2-thienyl)-2*E*,4*E*-hexadienamide (**11**, 18.1 mg) [32].

### 3.5. Determination of the Absolute Configuration of Capillinol (**2**)

Capillinol (11.5 mg, 0.067 mmoles) in 1 mL of dry CH_2_Cl_2_ at room temperature, 10.7 mg (0.064 mmoles) of (*R*)-(-)-α-methoxyphenylacetic acid (*R*-MPA), 14.2 mg (0.069 mmoles) of *N*, *N’*-dicyclohexylcarbodiimide (DCC), and 6.5 mg (0.053 mmoles) of 4-(dimethylamine)pyridine (DMAP) were added. The reaction mixture was stirred for 30 min in an inert nitrogen atmosphere. After this time, the solvent was removed under vacuum, and the reaction mixture was purified by column chromatography (CC) using a hexane/ethyl acetate polarity gradient (9:1 to 8:2) as the mobile phase, yielding 15.2 mg (74.2%) of capillinol *R*-(-)-α-methoxyphenylacetate as a colorless solid. Subsequently, the product was chelated with Ba (II), using a barium perchlorate salt dissolved in CH_3_CN, which was added until the solution was saturated to ensure its chelation. The ^1^H NMR shift differences between before and after suturing with Ba (II) were analyzed.

NMR data before Ba^2+^ chelation: NMR ^1^H (CDCl_3_, 600 MHz) δ 1.94 (3H, s, H-6), 3.37 (3H, s, OCH_3_), 4.89 (1H, s, H-1″), 6.47 (1H, s, H-1), 7.38–7.45 (8H, m, H-3′, H-4′, H-5′, H-4″, H-5″, H-6″, H-7″, H-8″), 7.47 (2H, dd, *J* = 2.1 Hz, *J* = 6.6 Hz, H-2′, H-6′); NMR ^13^C (CDCl_3_, 150 MHz) δ 4.15 (q, C-6), 57.8 (q, 2″, OCH_3_), 63.3 (s, C-4), 67.4 (s, C-1), 71.5 (s, C-2), 73.1 (s, C-3), 81.0 (s, C-5), 83.0 (s, C-1″), 128.2 (2xd, C-4″, C-8″), 128.4 (2xd, C-2′, C-6′), 129.6 (2xd, C-5″, C-7″), 129.7 (d, C-6″), 129.8 (2xd, C-2′, C-6′), 130.3 (d, C-3′), 137.0 (s, C-1′), 137.2 (s, C-3″).

NMR data after Ba^2+^ chelation: NMR ^1^H (CDCl_3_, 600 MHz) δ 1.94 (3H, s, H-6), 3.34 (3H, s, OCH_3_), 5.00 (1H, s, H-1″), 6.51 (1H, s, H-1), 7.38–7.45 (2H, m, H-2′, H-6′), 7.47 (8H, dd, *J* = 2.1 Hz, *J* = 6.6 Hz, H-3′, H-4′, H-5′, H-4″, H-5″, H-6″, H-7″, H-8″); NMR ^13^C (CDCl_3_, 150 MHz) δ 4.15 (q, C-6), 57.8 (q, OCH_3_), 63.3 (s, C-4), 67.4 (s, C-1), 71.5 (s, C-2), 73.1 (s, C-3), 81.0 (s, C-5), 83.0 (s, C-1″), 128.2 (2xd, C-4″, C-8″), 128.4 (2xd, C-2′, C-6′), 129.6 (2xd, C-5″, C-7″), 129.7 (d, C-6″), 129.8 (2xd, C-2′, C-6′), 130.3 (d, C-3′), 137.0 (s, C-1′), 137.2 (s, C-3″).

### 3.6. Biological Assays

#### 3.6.1. Fungal Culture

Phytopathogenic fungi, *Alternaria alternata*, *Botrytis cinerea*, and *Fusarium oxysporum*, were maintained at 25 °C in darkness, and periodically replicated in Petri dishes with PGA culture medium and tetracycline to avoid contamination. Strains of *B. cinerea* (B05.10) and *A. alternata* (Aa 100) were isolated from *Vitis vinifera* and *Lycopersicon esculentum*, respectively, both supplied by the Universidad de La Laguna, Tenerife. *F. oxysporum f.* sp. *lycopersici* (2715) strain, isolated from *Lycopersicon esculentum*, was provided by the Colección Española de Cultivos Tipo (CECT) from Valencia, Spain.

#### 3.6.2. In Vitro Test-Assay on Mycelium

The antifungal activity of extracts, fractions, subfractions, and isolated compounds was performed on the mycelium stage of *F. oxysporum*, *B. cinerea,* and *A. alternata* using an agar-dilution protocol as previously described [27]. Briefly, an aliquot of test sample in EtOH (50 mg/mL stock solution) was dissolved in warm PGA (final volume 5 mL) and placed in a 9 cm Petri dish. The medium containing the sample was allowed to solidify. Eight 4 mm diameter discs of each fungus were deposited in a Petri dish with 1 mg/mL final concentration of each tested and control sample and incubated for 48 h (*B. cinerea*) or 72 h (*A. alternata* and *F. oxysporum*). A Petri dish with eight discs was analyzed for each pathogen. Colony growth was measured with an image-processing software ImageJ 1.53e-Wayne Rasband (NIH) (ANOVA). The diameter of each colony was measured twice, making a cross, and the average of both measurements was taken. Percentage growth inhibition (%GI) was calculated using the equation: %GI = [(C − T)/C] × 100, where C and T are the diameters of the control and test treated colonies, respectively. Fractions and subfractions exhibiting %GI higher than 20% at 1 mg/mL were also evaluated at 0.5 and 0.1 mg/mL doses. Pure compounds growth inhibition assays were conducted at 0.1, 0.05 and 0.01 mg/mL. Fosbel-Plus and/or Ortiva PC, and EtOH were used as positive and negative controls, respectively.

#### 3.6.3. Statistical Analysis

All data were shown as a mean ± standard deviation (SD). The percentage of inhibition was analyzed using one-way analysis of variance (ANOVA) [41]. Prior to analysis, data were assessed for normality and homogeneity of variances using the Shapiro–Wilk and Levene’s tests, respectively, to ensure the validity of ANOVA assumptions. Tukey’s HSD multiple comparisons of means was used to compare concentrations and treatments. Differences of *p* < 0.05 were considered statistically significant. All analyses were performed using Social Science Statistics, 2018.

## 4. Conclusions

This research pointed out the potential of *A. frutescens* as a sustainable and competitive alternative to conventional pesticides against high-risk phytopathogenic fungi, fostering more ecological and efficient agricultural practices. It overcame significant cultivation challenges and confirmed the preservation of phytochemical and fungicidal profiles in cultivated plants, offering a sustainable alternative source that could mitigate wild-type collection pressure. The bioassay-guided fractionation of the ethanolic extracts successfully identified polyacetylenes, notably capillin, capillinol, and frutescinone exhibiting an outstanding antifungal profile, comparable to that of the commercial pesticides used as positive controls. These results highlight the potential of these compounds as fungicides that could be applied in combination with other biological agents for integrated crop disease management. Furthermore, these compounds could serve as a versatile platform through structural optimization for the development of more effective fungicides. Nonetheless, further investigations are warranted to elucidate their mode of action, and to optimize the experimental extraction process for application in sustainable, environmentally safe, and economically viable integrated crop protection management.

## Figures and Tables

**Table 1:** Antifungal effects (% growth inhibition) of compounds **1**–**11** at 0.1–0.01 mg/mL from *Argyranthemum frutescens* roots against *Alternaria alternata*, *Botrytis cinerea,* and *Fusarium oxysporum*.

Sample	*A. alternata*			*B. cinerea*			*F. oxysporum*		
	0.1	0.05	0.01	0.1	0.05	0.01	0.1	0.05	0.01
**1**	34.6 ± 3.3	31.7 ± 2.2	22.6 ± 2.5	76.3 ± 5.4	58.4 ± 6.54	23.6 ± 7.6	36.2 ± 4.2	34.0 ± 3.2	14.4 ± 3.0
**2**	66.3 ± 2.3	35.3 ± 3.4	24.7 ± 4.6	95.8 ± 3.2	60.0 ± 5.4	NA	83.6 ± 3.8	44.5 ± 4.9	18.2 ± 3.8
**3**	25.5 ± 3.8	NA	ND	100.0 ± 0.0	92.7 ± 7.1	39.9 ± 6.8	94.9 ± 1.4	84.4 ± 11.7	NA
**4**	56.6 ± 4.1	55.4 ± 2.4	37.9 ± 3.2	61.8 ± 8.9	60.9 ± 7.4	21.1 ± 5.9	23.9 ± 5.2	17.4 ± 2.5	NA
**5**	48.1 ± 4.2	40.4 ± 4.9	13.3 ± 5.2	90.8 ± 4.1	90.8 ± 2.3	70.6 ± 4.5	45.6 ± 6.3	31.8 ± 4.7	NA
**6**	42.6 ± 6.8	28.2 ± 4.2	NA	85.1 ± 3.2	73.2 ± 2.6	32.2 ± 3.1	23.9 ± 2.4	NA	ND
**7**	39.3 ± 2.3	38.4 ± 1.9	13.8 ± 2.9	39.7 ± 4.6	37.4 ± 5.6	20.2 ± 5.8	19.5 ± 1.5	ND	ND
**8**	NA	ND	ND	NA	ND	ND	18.9 ± 2.5	ND	ND
**9**	71.3 ± 2.7	45.3 ± 2.9	33.4 ± 6.6	90.2 ± 5.5	80.6 ± 2.2	57.4 ± 3.0	41.7 ± 1.9	12.7 ± 5.9	ND
**10**	29.3 ± 2.1	10.7 ± 3.3	NA	70.1 ± 6.6	70.0 ± 6.1	23.7 ± 4.1	20.6 ± 2.0	18.7 ± 8.1	ND
**11**	40.8 ± 2.9	NA	ND	42.5 ± 2.5	38.5 ± 4.5	17.2 ± 5.2	43.4 ± 0.5	13.9 ± 4.7	ND
**FP**	74.2 ± 4.8	65.5 ± 4.0	38.1 ± 3.8	23.6 ± 4.8	21.9 ± 3.8	13.5 ± 3.6	78.8 ± 4.7	44.4 ± 4.8	28.7 ± 2.5
**A**	30.4 ± 2.1	31.2 ± 4.0	33.4 ± 1.9	67.8 ± 4.1	50.0 ± 7.3	32.6 ± 6.1	53.2 ± 5.1	51.6 ± 5.1	32.2 ± 6.2

The values are expressed as percentage of growth inhibition (%GI) relative to control growth in the absence of inhibitory agents (negative control). Compounds with a GI higher than 20% at 0.1 mg/mL were assayed at lower concentrations (0.05 and 0.01 mg/mL). NA: Non-active (% GI ˂ 10%). ND: not determined. FP: Fosbel-Plus and A: Azoxystrobin were used as positive controls. The data shown are the average of eight independent experiments ± SD (standard deviation).

## Data Availability

Data are contained within the article and Supplementary Materials.

## Supplementary Materials

The following supporting information can be downloaded at: https://www.mdpi.com/article/10.3390/plants14070985/s1, Experimental part S1. Bioguided fractionation: Extraction and Isolation; Figures S1–S11: ^1^H and ^13^C NMR spectra of metabolites **1**–**11** isolated from wild and cultivated Argyranthemum frutescens roots; Figure S12: ^1^H NMR spectra of capillinol (*R*)-α-methoxyphenylacetate before and after saturation with barium (II) salt as the chelating agent; Figure S13: representative photographs of fungal growth inhibition in a dilution agar assay; Table S1: antifungal effects (% growth inhibition) of extract, fractions, and subfractions from roots of wild Argyranthemum frutescens against *Alternaria alternata*, *Botrytis cinerea,* and *Fusarium oxysporum*; Table S2: antifungal effects (% growth inhibition) of extract, fractions, and subfractions from roots of cultivated *Argyranthemum frutescens* against *Alternaria alternata*, *Botrytis cinerea,* and *Fusarium oxysporum*.
