# Supplementary material for: Eco-Friendly Crop Protection: Argyrantemum frutescens, a Source of Biofungicides
Source: Plants (Basel). 2025 Mar 21;14(7):985. doi: 10.3390/plants14070985 (PMC11990897; doi:10.3390/plants14070985)
Supplement: Supplementary file 1 [file plants-14-00985-s001.zip › plants-3526180-supplementary.pdf]

Electronic Supporting Information

Eco-Friendly Crop Protection: *Argyranthemum frutescens*, a Source of Biofungicides

Eduardo Hernández-Álvarez[1], Samuel Rodríguez-Sabina[2], Noelia Labrador-García[1], Javier Hernández Pérez[1], Carolina P. Reyes[3], María Ángeles Llaría-López[4], Ignacio A. Jiménez[1] and Isabel L. Bazzocchi[1,*]

[1] Instituto Universitario de Bio-Orgánica Antonio González, and Departamento de Química Orgánica, Universidad de La Laguna, Avenida Astrofísico Francisco Sánchez 2, 38206 La Laguna, Tenerife, Spain.
[2] Departamento de Botánica, Ecología y Fisiología Vegetal, Universidad de La Laguna, Avenida Astrofísico Francisco Sánchez, 38206 La Laguna, Tenerife, Spain.
[3] Instituto Universitario de Bio-Orgánica Antonio González, and Departamento de Bioquímica, Microbiología, Biología Celular y Genética, Universidad de La Laguna, Avenida Astrofísico Francisco Sánchez 2, 38206 La Laguna, Tenerife, Spain.
[4] Área de Gestión del Medio Natural y Seguridad, Cabildo Insular de Tenerife, C/ Las Macetas s/n, Pabellón Insular Santiago Martín, 38108 La Laguna, Tenerife, Spain.

Table of Contents

Experimental Part S1. Bioguided fractionation**:** Extraction and Isolation.

Figures S1-S11. ^{1}H and ^{13}C NMR spectra of metabolites **1-11** isolated from wild and cultivated *Argyranthemum frutescens* roots.

Figure S12. ^{1}H NMR spectra of capillinol (*R*)-(-)-α-methoxyphenylacetate before and after saturation with barium (II) salt as the chelating agent.

Figure S13. Representative photographs of fungal growth inhibition in a dilution agar assay.

Table S1. Antifungal effects (% Growth Inhibition) of extract, fractions, and sub-fractions from roots of wild *Argyranthemum frutescens* against *Alternaria alternata*, *Botrytis cinerea* and *Fusarium oxysporum.*

Table S2. Antifungal effects (% Growth Inhibition) of extract, fractions, and sub-fractions from roots of cultivated *Argyranthemum frutescens* against *Alternaria alternata*, *Botrytis cinerea* and *Fusarium oxysporum.*

Experimental part S1. Bioguided fractionation: Extraction and isolation

Extraction and isolation of wild-type Argyranthemum frutescens.

The air-dried and powdered roots of *A. frutescens* (L.) Sch. Bip. Subsp. frutescens (450.9 g) were extracted by maceration with 96% EtOH (45 L x 3 times) at room temperature for 24 h for each maceration process and concentrated under reduced pressure to yield 17.9 g of residue (4.0%), which was assayed on phytopathogenic fungi (*Fusarium oxysporum*, *Botrytis cinerea*, and *Alternaria alternata*) giving a potent activity. Therefore, the ethanolic extract was suspended in water (H_2O) and solvent-solvent partitioned sequentially with hexanes (Hx) and ethyl acetate (EtOAc). The organic phases were concentrated under reduced pressure to give Hx (7.7 g) and EtOAc (3.8 g) fractions, whereas the aqueous residue was lyophilized providing the H_2O fraction (4.0 g). Biological evaluation revealed that the organic fractions were active against the phytopathogenic fungi and were further investigated.

The most active hexane fraction (7.7 g) was chromatographed on a silica gel column, using mixtures of hexane/EtOAc of increasing polarity (10:0 to 0:10) as eluent to afford fourteen sub-fractions, which were combined based on their TLC profile in sub-fractions A1-A8. The most active sub-fraction A2 (3.5 g) was chromatographed on a silica gel column, using mixtures of hexane/EtOAc of increasing polarity (10:0 to 9:1) as eluent to afford fifty-three sub-fractions, which were combined based on their TLC profile in sub-fractions A2.1-A2.9. Sub-fraction A2.5 (120.2 mg) was further purified on Sephadex LH-20 (hexanes/$CHCl_3$/MeOH, 2:1:1), to afford fifty sub-fractions, which were combined based on their TLC profile in sub-fractions A2.5A-A2.5F. Sub-fractions A2.5B (50.1 mg), A2.5C (16.8 mg) and A2.5D (13.6 mg) were identified as compound **1**, capillinol acetate. Sub-fraction A3 (1.9 g) was purified by Sephadex LH-20 (hexanes/$CHCl_3$/MeOH, 2:1:1) and combined based on their TLC profile to afford eleven sub-fractions A3.C1-A3.C11. Sub-fractions A3.C4 (50.1 mg), A3.C5 (16.8 mg) and A3.C6 (13.6 mg) were identified as compound **1**, capillinol acetate. Sub-fraction A4 (1.8 g) was purified by Sephadex LH-20 (hexanes/$CHCl_3$/MeOH, 2:1:1) and combined based on their TLC profile to afford ten sub-fractions A4.D1-A4.D10. Sub-fraction A4.D9 (62.5 mg) was chromatographed on a silica gel column, using mixtures of hexane/dichloromethane of increasing polarity (5:5 to 0:10)

as eluent and combined based on their TLC profile in sub-fractions A4.D9A-A4.D9E. Sub-fraction A4.D9C (19.6 mg) was identified as compound **2**, capillinol. Sub-fraction A4.D7 (219.5 mg) was chromatographed on a silica gel column, using mixtures of hexane/dichloromethane of increasing polarity (6:4 to 0:10) as eluent and combined based on their TLC profile in sub-fractions A4.D7A-A4.D7J. Sub-fractions A4.D7B (6.4 mg) and A4.D7H (11.4 mg) were identified as compound **3** (capillin) and **6** (frutescin), respectively. Sub-fraction A4.D7E (55.3 mg) was further purified by preparative TLC (dichloromethane) to give compound **5** (frutescinone, 13.1 mg). Sub-fraction A5 (0.7 g) was purified by Sephadex LH-20 (hexane/$CHCl_3$/MeOH, 2:1:1) to afford twenty-seven sub-fractions, which were combined based on their TLC profile to afford sub-fractions A5.E1-A5.E5. Sub-fraction A5.E2 (118.6 mg) was further purified by preparative TLC (dichloromethane: acetone, 10:0.5) to give compound **8** (2*E*,4*E*-tetradecadienoic acid isobutyl amide, 7.6 mg). Sub-fraction A6 (1.16 g) was purified by Sephadex LH-20 (hexane/$CHCl_3$/MeOH, 2:1:1) to afford fifty sub-fractions, which were combined based on their TLC profile to afford sub-fractions A6.F1-A6.F6. Sub-fraction A6.F3 (103.5 mg) was chromatographed on a silica gel column, using mixtures of hexane/dichloromethane of increasing polarity (10:0 to 10:0.5) as eluent and combined based on their TLC profile in sub-fractions A6.F3A-A6.F3G. Sub-fraction A6.F3A (11.2 mg) was further purified by preparative TLC (hexanes: ethyl acetate, 5:5) to give compound **4** (frutescinol acetate, 4.6 mg). Sub-fraction A6.F3E (8.1 mg) was further purified by preparative TLC (hexane: ethyl acetate, 5:5) to give compound **7** [*N*-isobutyl-6-(2-thienyl)-2*Z*,4*Z*-hexadienamide, 1.6 mg)].

Extraction and isolation of cultivated Argyranthemum frutescens.

The most active fractions against phytopathogenic fungi, that corresponds to the hexane residue (13.5 g) was chromatographed on a silica gel column, using mixtures of hexane/EtOAc of increasing polarity (10:0 to 0:10) as eluent to afford ten subfractions, which were combined based on their TLC profile in subfractions B1-B7. Phytopathogenic fungi activity revealed that subfractions B1-B5 were active against the strains of *A. alternata*, *B. cinerea* and *F. oxysporum*. Subfractions were subjected to several chromatography steps until obtaining the pure compounds.

Therefore, sub-fraction B1 (2.2 g) was chromatographed on Sephadex LH-20 (hexane/$CHCl_3$/MeOH, 2:1:1), affording subfractions B1.1-B1.7. Sub-fraction B1.5 (890 mg) was chromatographed on a silica gel column, using mixtures of hexane/EtOAc of increasing polarity (10:0 to 8:2). to give compounds **1** (216.8 mg) and **4** (5.1 mg). Subfractions B1.4 and B1.6 were identified as compound **1** (243.1 mg) and **9** (153.5 mg), respectively, by 1D and 2D NMR analysis. Sub-fraction B2 (1.2 g) was chromatographed on Sephadex LH-20 (hexane/$CHCl_3$/MeOH, 2:1:1), affording 24 subfractions, which were combined based on their TLC profile in subfractions B2.1-B2.12. Sub-fraction B2.9 (152.5 mg) was chromatographed on a silica gel column, using mixtures of hexane/EtOAc of increasing polarity (10:0 to 9:1) to afford 69 subfractions, which were combined based on their TLC profile in B2.9A-B2.9C subfractions. B2.9A was further purified by silica gel column, using mixtures of hexane/CH_2Cl_2 of increasing polarity (7:3 to 1:1) to give compound **6** (8.8 mg). B2.9B and B2.9C subfractions were identified as compound **4** (67.5 mg) and **11** (34.5 mg), respectively. Subfraction B2.11 (91.8 mg) was chromatographed on a silica gel column, using mixtures of hexane/EtOAc of increasing polarity (10:0 to 9:1) to afford 9 subfractions, which were combined based on their TLC profile in B2.11A-B2.11C. C11B was purified by preparative TLC (hexane/CH_2Cl_2) (7:3) to give compound **10** (7.5 mg). B2.11A was identified as compound **9** (9.6 mg) through 1D and 2D-NMR analysis. Subfractions B2.7 and B2.12 were identified as compound **1** (207.5 mg) and **2** (22.6 mg) respectively. Sub-fraction B3 (480.6 mg) was chromatographed on Sephadex LH-20 (hexane/$CHCl_3$/MeOH, 2:1:1), affording 21 subfractions which were combined based on their TLC profile in subfractions B3.1-B3.4. B3.4 was identified as compound **11** (34.2 mg) by 1D and 2D NMR analysis. B3.3 was purified by preparative TLC (benzene/EtOAc) (7:3) to give compound **11** (4.6 mg) according to the NMR analysis. Sub-fraction B4 (699.2 mg) was chromatographed on Sephadex LH-20 (hexane/$CHCl_3$/MeOH, 2:1:1), affording 24 subfractions, which were combined based on their TLC profile in subfractions B4.1-B4.8. B4.7 (87.8 mg) was chromatographed on a silica gel column, using mixtures of hexane/EtOAc of increasing polarity (9:1 to 6:4) to afford 48 subfractions, which were combined based on their TLC profile in subfractions B4.7A-B4.7G. B4.7G was purified by Sephadex LH-20 chromatography (hexane/$CHCl_3$/MeOH, 2:1:1) identifying compound **11** (6.6 mg). B4.7C and B4.7E subfractions were identified as compound **6** (24.1 mg and 2.7 mg,

respectively) by 1D and 2D NMR analysis. B4.3 and B4.5 were identified as compounds **11** (12.4 mg) and **6** (38.2 mg), respectively, by 1D and 2D NMR analysis. Sub-fraction B5 (1.33 g) was chromatographed on Sephadex LH-20 (hexane/$CHCl_3$/MeOH, 2:1:1), affording 25 subfractions, which were combined based on their TLC profile in subfractions B5.1-B5.7. The sub-fraction B5.3 (313.1 mg) was chromatographed on a silica gel column, using mixtures of hexane/EtOAc of increasing polarity (10:0 to 7:3) to afford 10 subfractions, which were combined based on their TLC profile in subfractions B5.3A and B5.3B. B5.3A was chromatographed on a silica gel column, using mixtures of hexane/CH_2Cl_2 of increasing polarity (6:4 to 4:6) to yield compounds **4** (6.1 mg) and **10** (1.2 mg), whereas B5.3B was identified as compound **6** (156.0 mg) by 1D and 2D NMR analysis. B5.2 (398.4 mg) was chromatographed on a silica gel column, using mixtures of hexane/EtOAc of increasing polarity (10:0 to 7:3) to afford 20 subfractions, which were combined based on their TLC profile in subfractions B5.2A-B5.2F. B5.2E and B5.2F were identified as **8** (18.1 mg) and **6** (39.8 mg), respectively. Subfractions B5.4 and B5.7 were identified as compound **11** (381.4 mg) and **2** (47.0 mg), respectively.

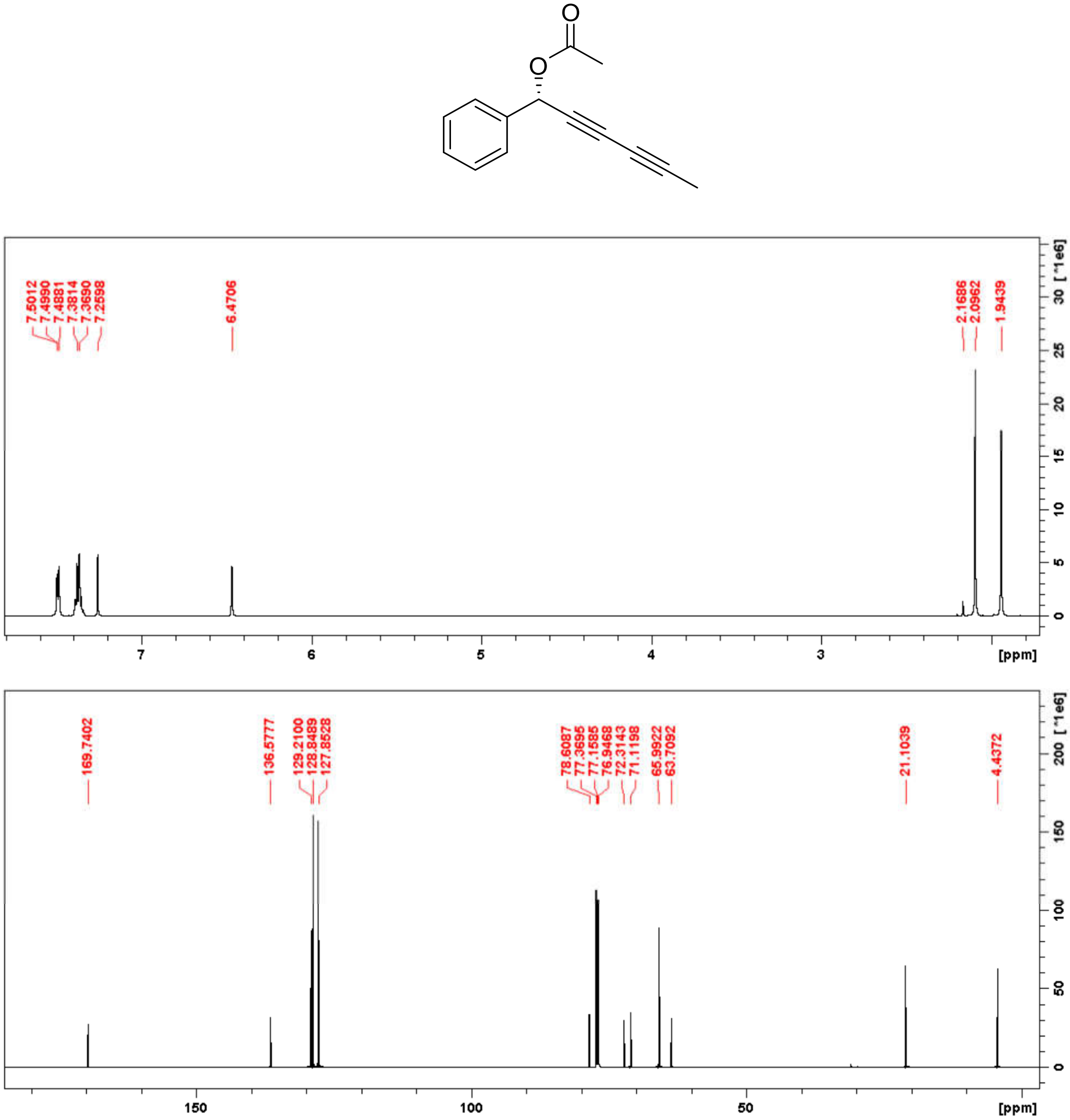

Figure S1. ^{1}H and ^{13}C NMR spectra of **1** (capillinol acetate) in $CDCl_3$ (500 and 125 MHz, respectively).

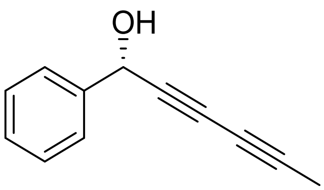

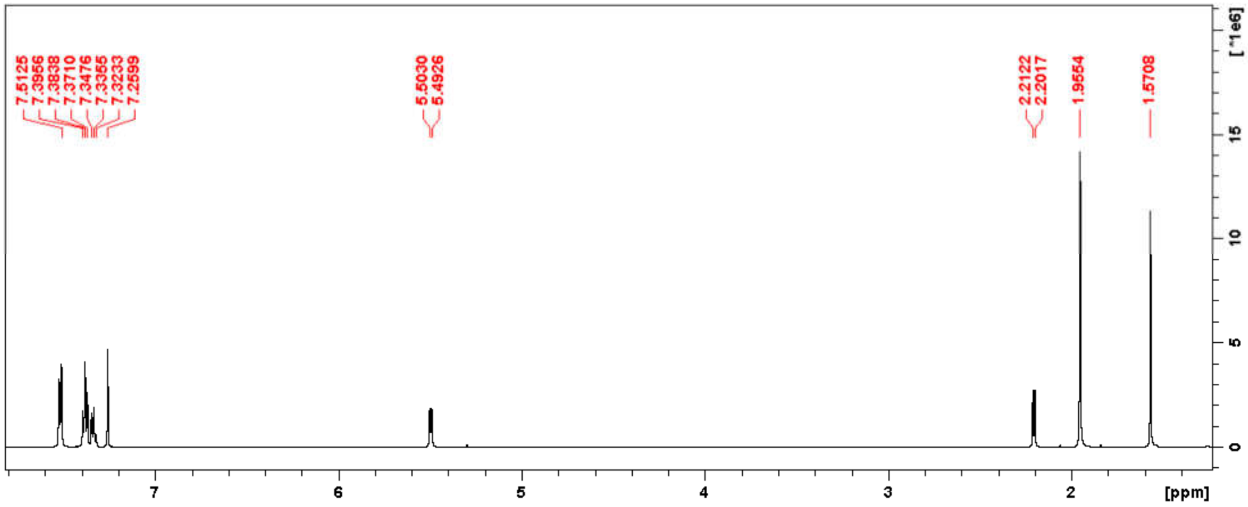

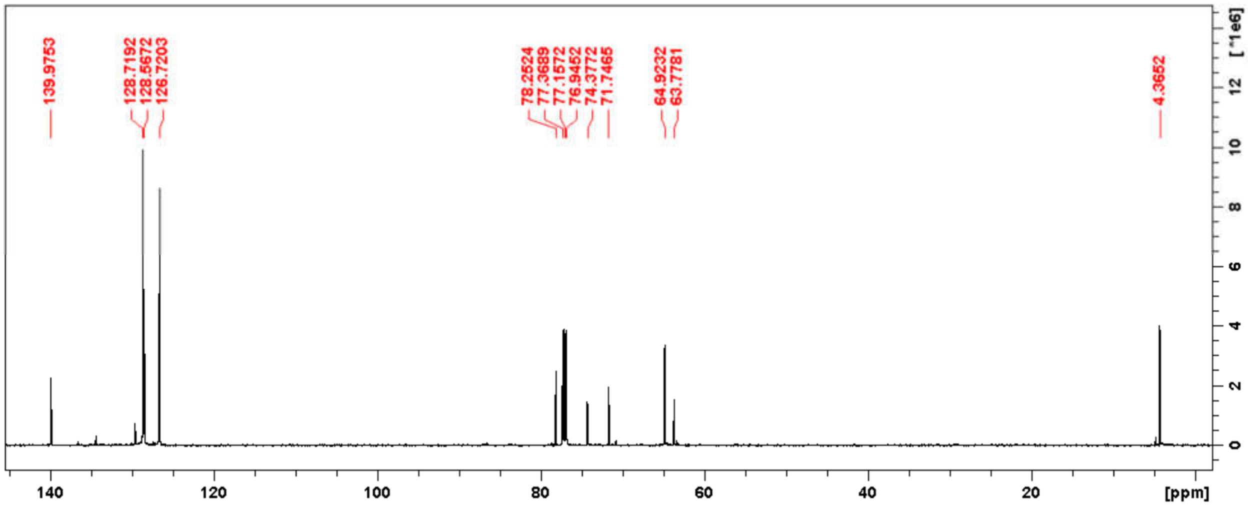

Figure S2. ^{1}H and ^{13}C NMR spectra of **2** (capillinol) in $CDCl_3$ (500 and 125 MHz, respectively).

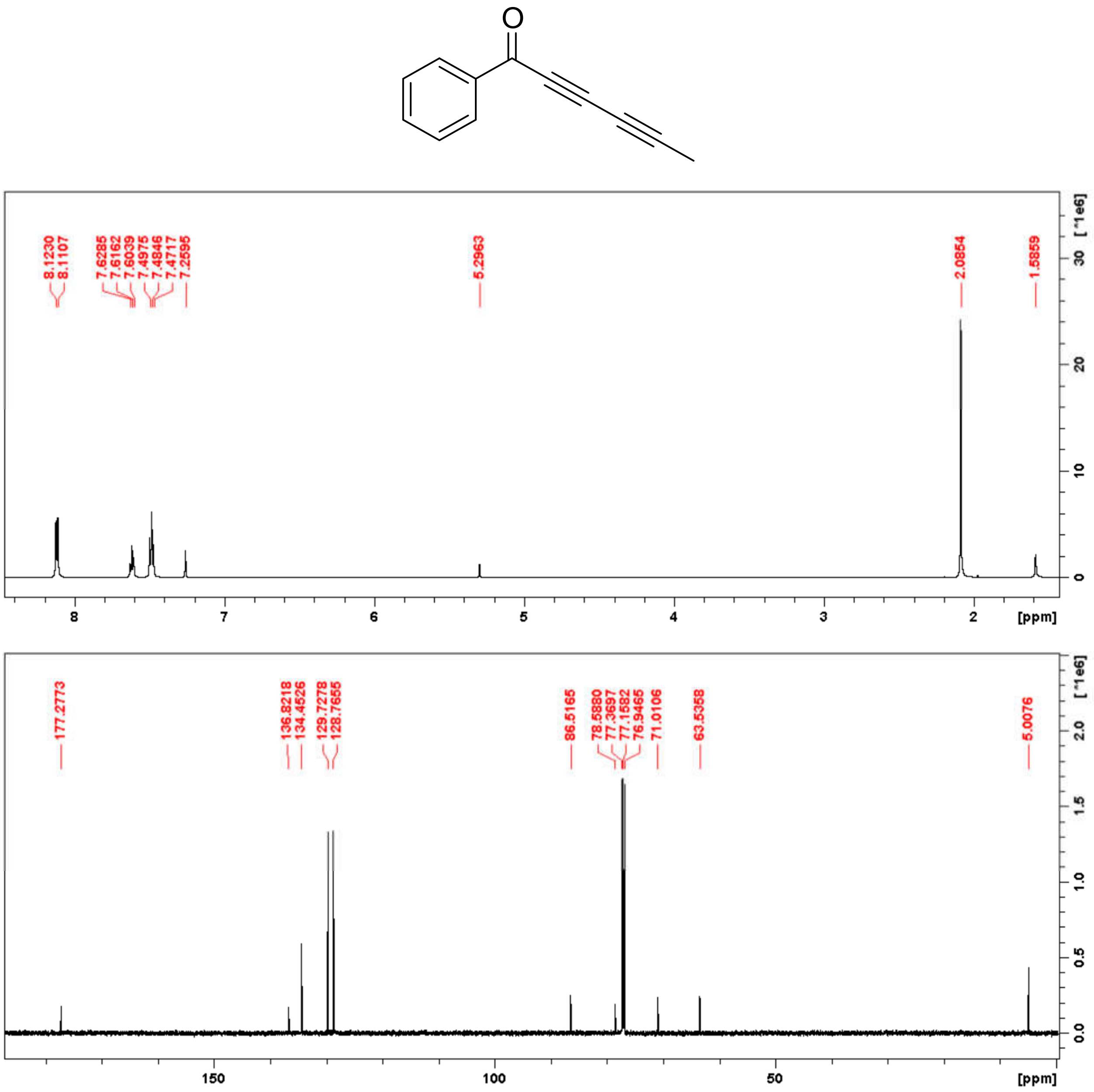

Figure S3. ^{1}H and ^{13}C NMR spectra of **3** (capillin) in $CDCl_3$ (500 and 125 MHz, respectively).

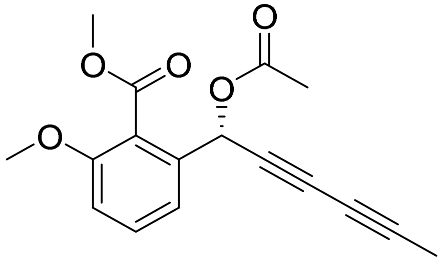

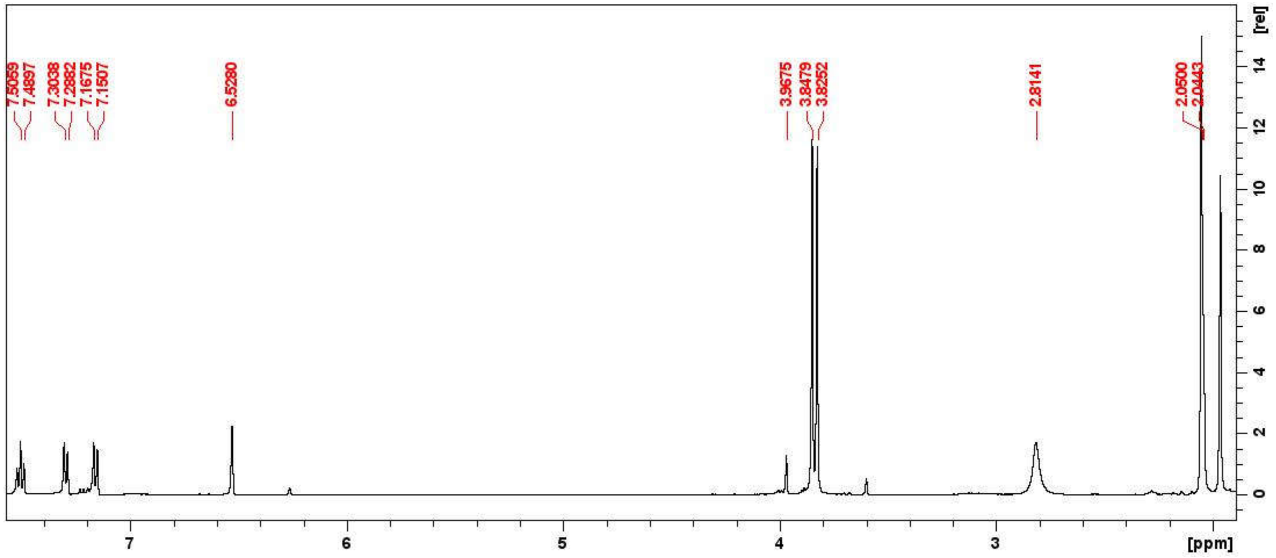

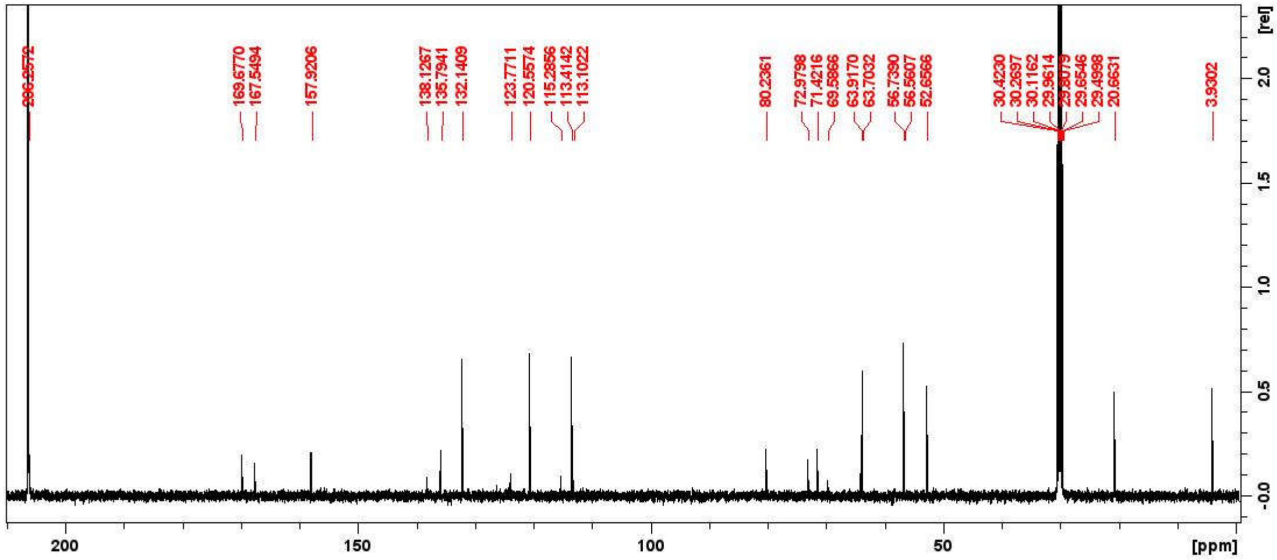

Figure S4. ^{1}H and ^{13}C NMR spectra of **4** (frutescinol acetate) in $CDCl_3$ (500 and 125 MHz, respectively).

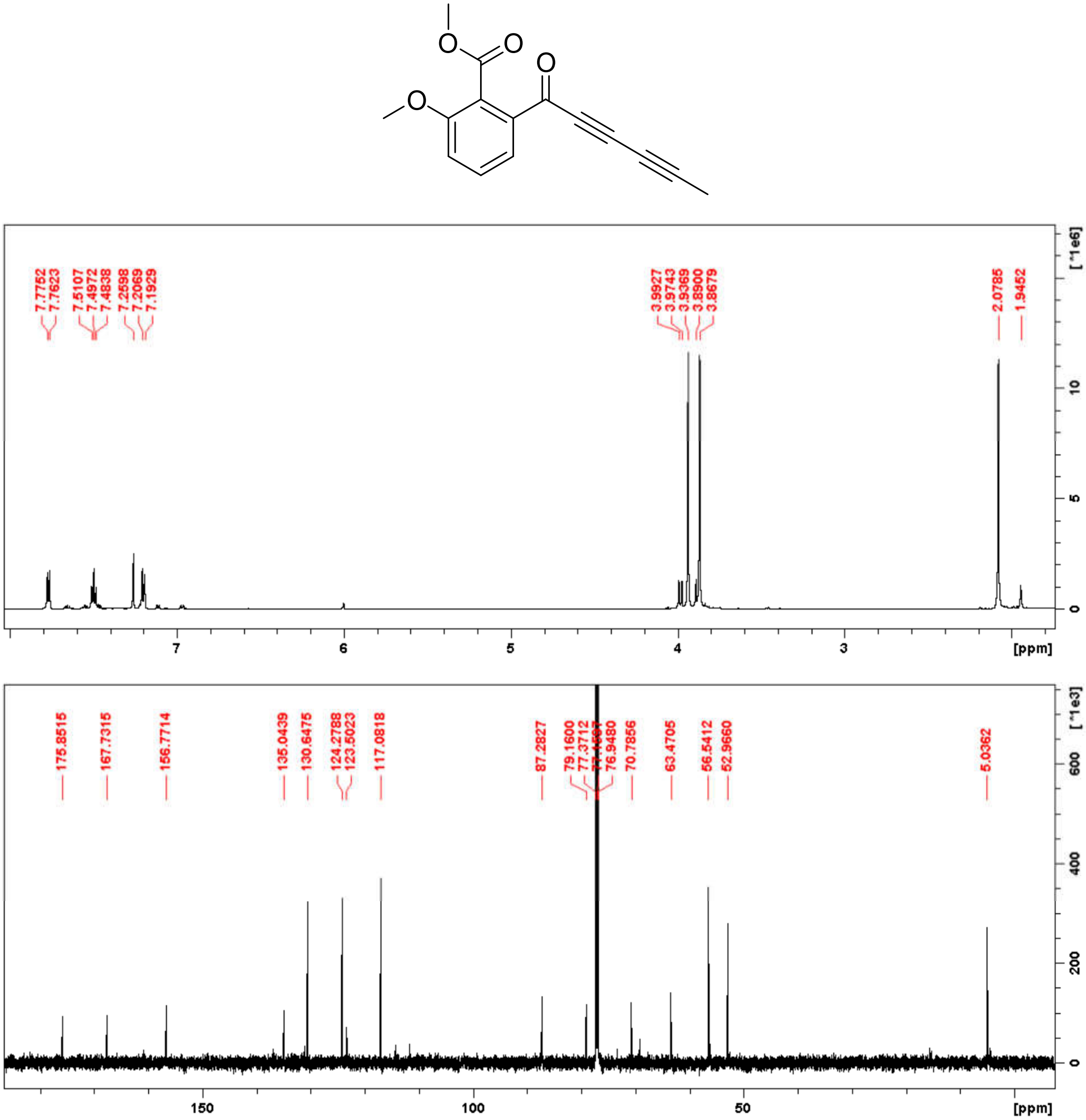

Figure S5. ^{1}H and ^{13}C NMR spectra of **5** (frutescinone) in $CDCl_3$. (600 and 150 MHz, respectively).

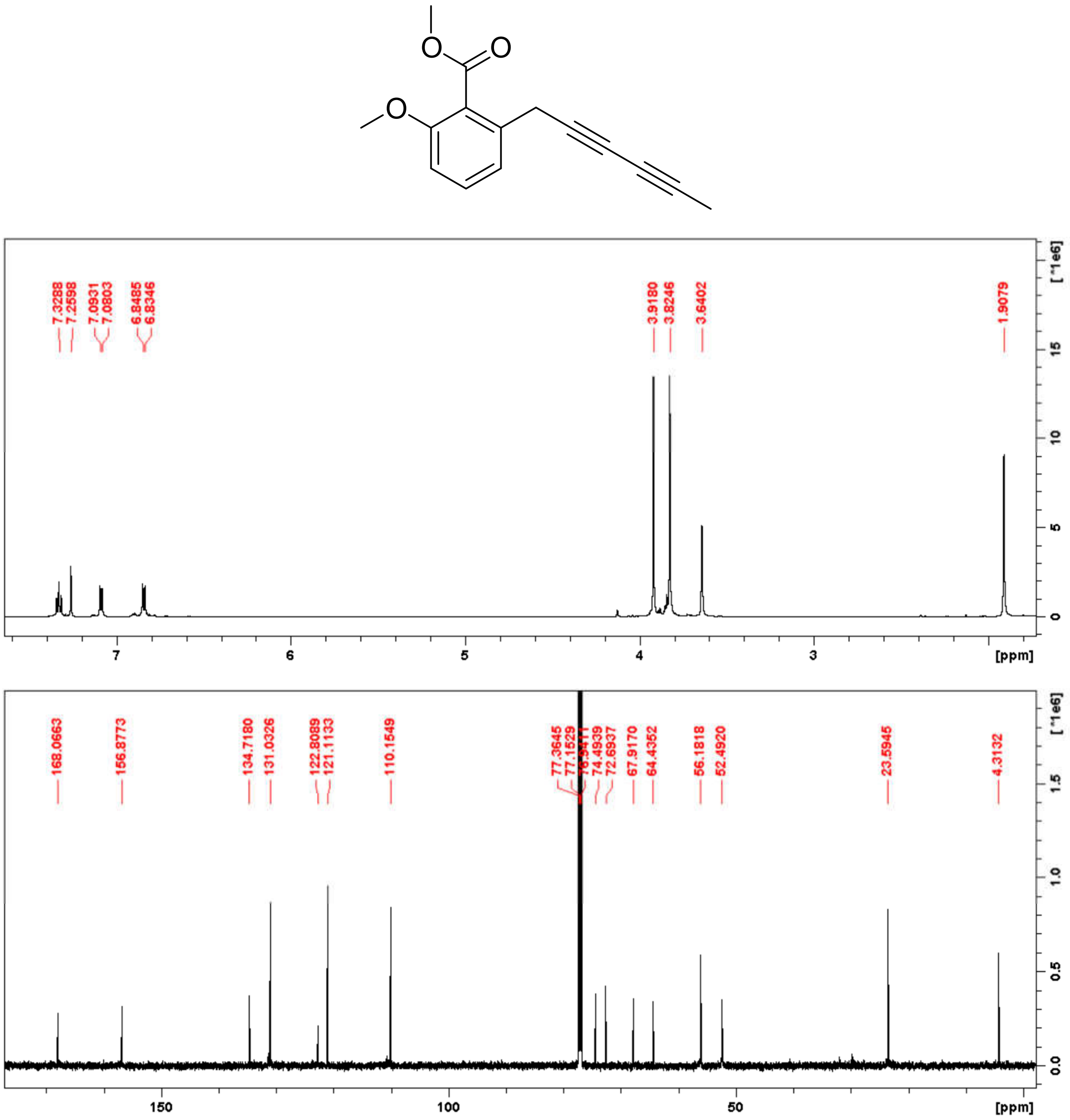

Figure S6. ^{1}H and ^{13}C NMR spectra of **6** (frutescin) in $CDCl_3$ (500 and 125 MHz, respectively).

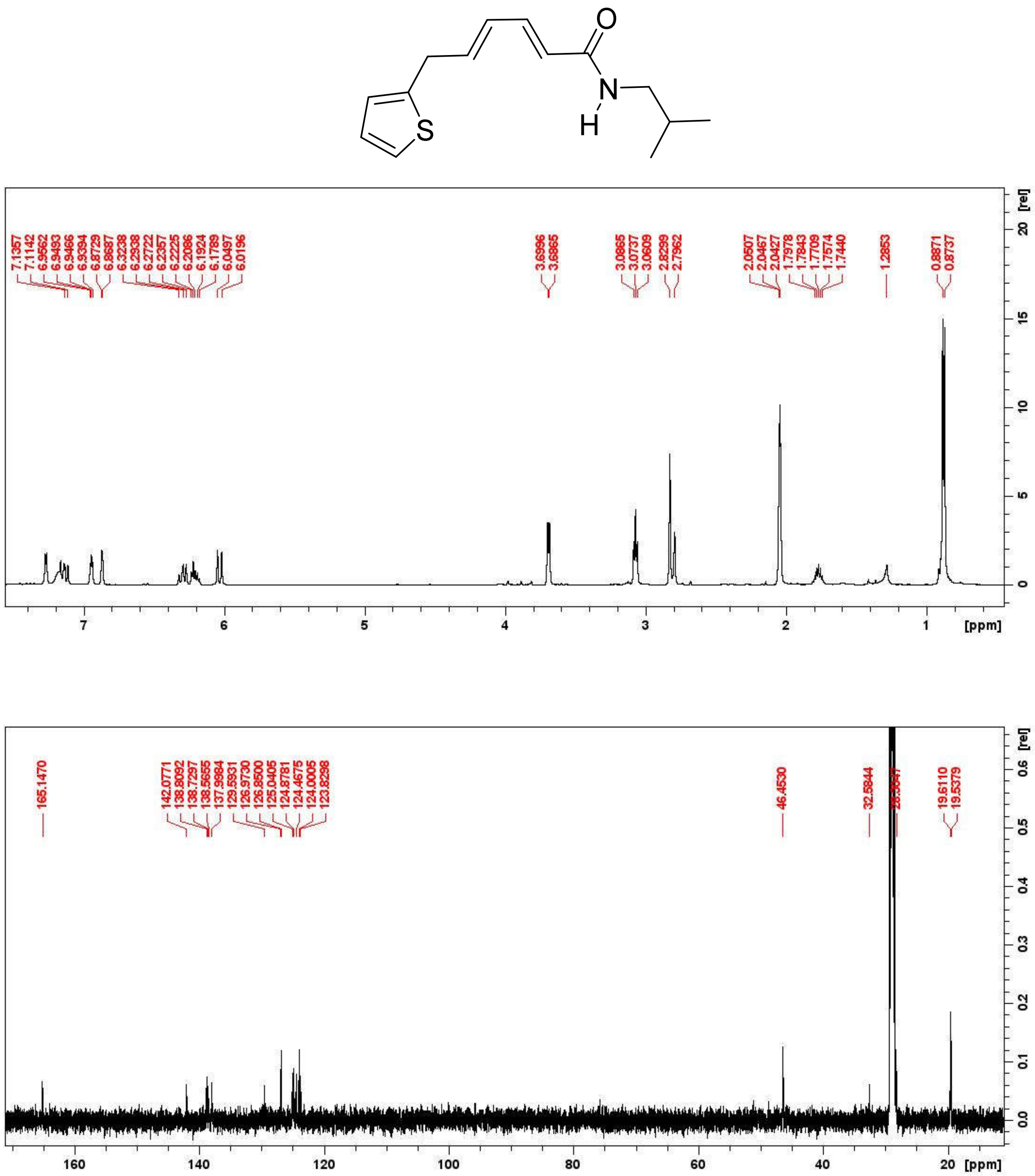

Figure S7. ^{1}H and ^{13}C NMR spectra of **7** [*N*-isobutyl-6-(2-thienyl)-2*Z*,4*Z*-hexadienamide] in $CDCl_3$ (500 and 125 MHz, respectively).

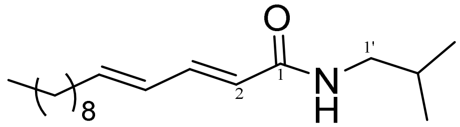

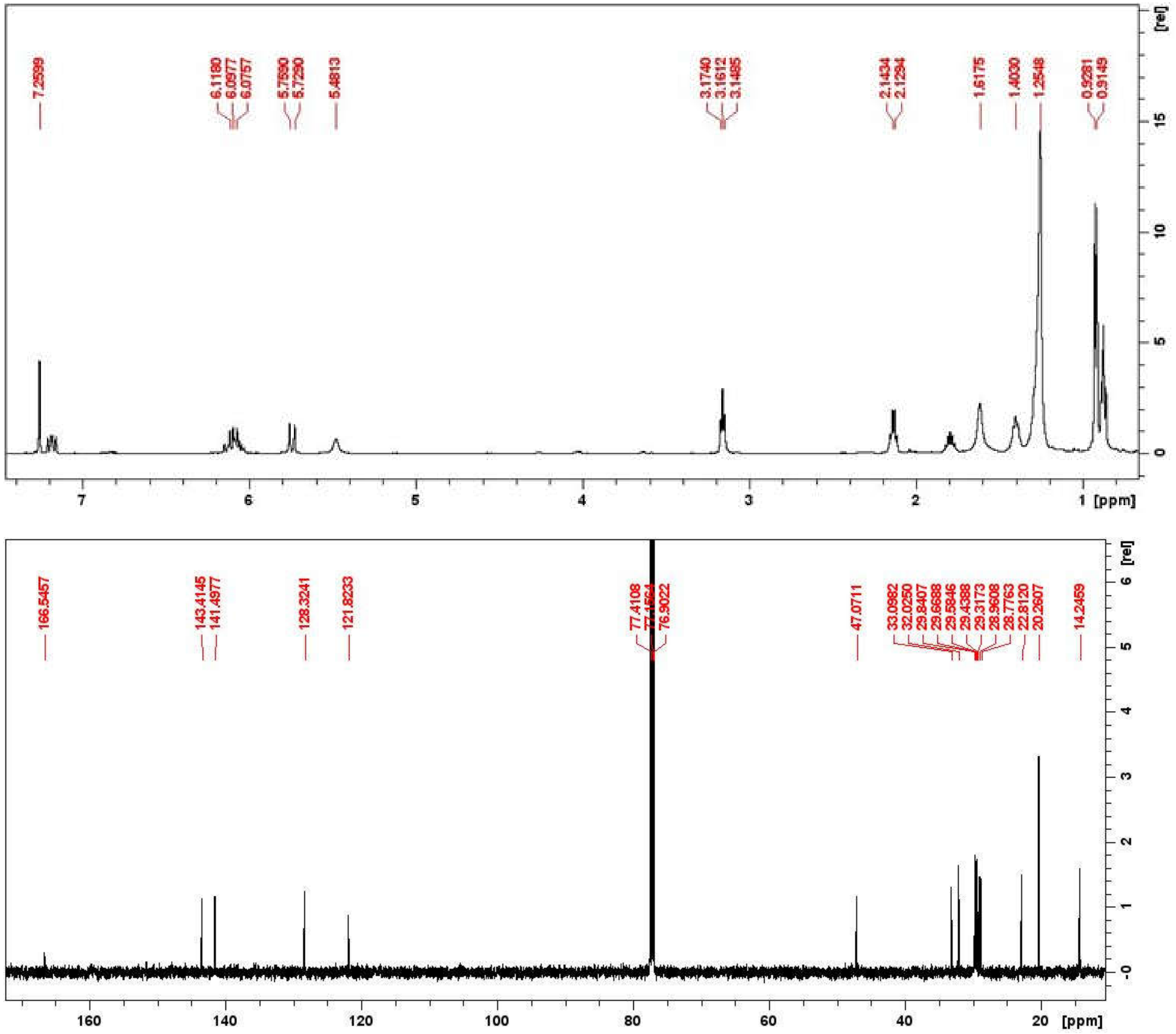

Figure S8. ^{1}H and ^{13}C NMR spectra of **8** (2*E*,4*E*-tetradecadienoic acid isobutyl amide) in $CDCl_3$ (500 and 125 MHz, respectively).

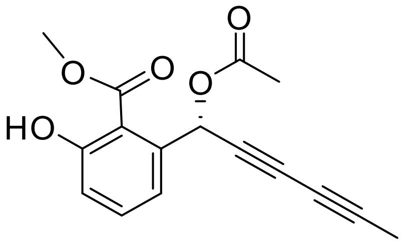

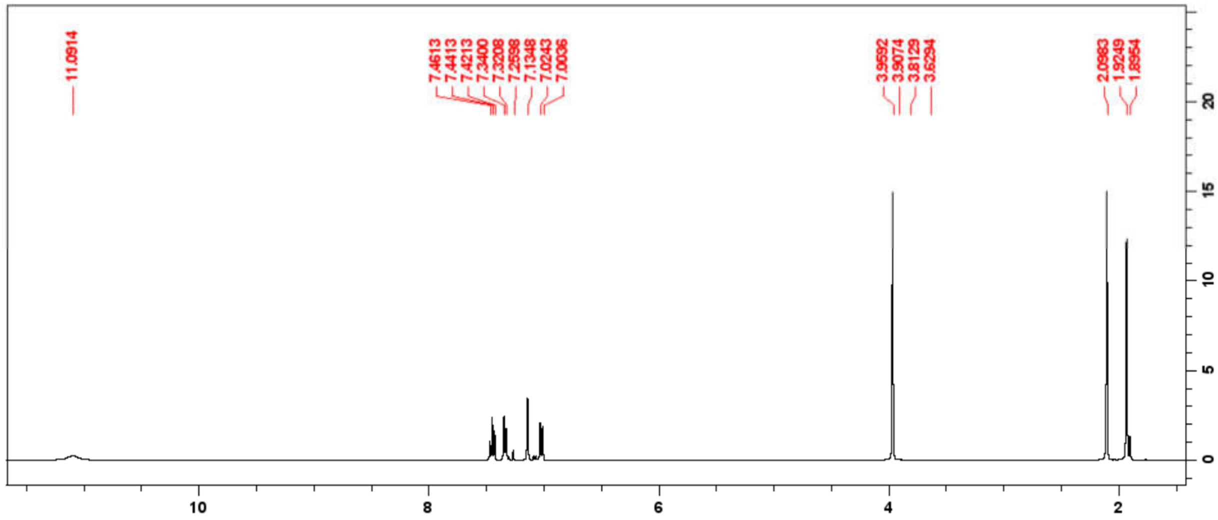

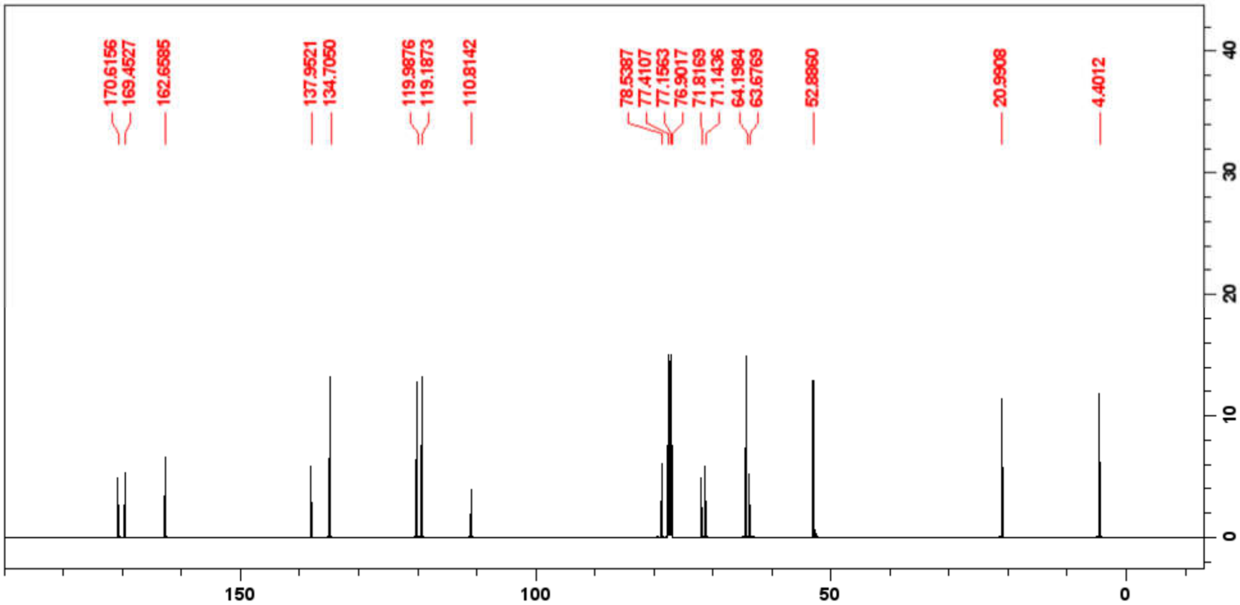

Figure S9. ¹H and ¹³C NMR spectra of **9** (3′-*O*-demethylfrutescinol acetate) in $CDCl_3$ (500 and 125 MHz, respectively).

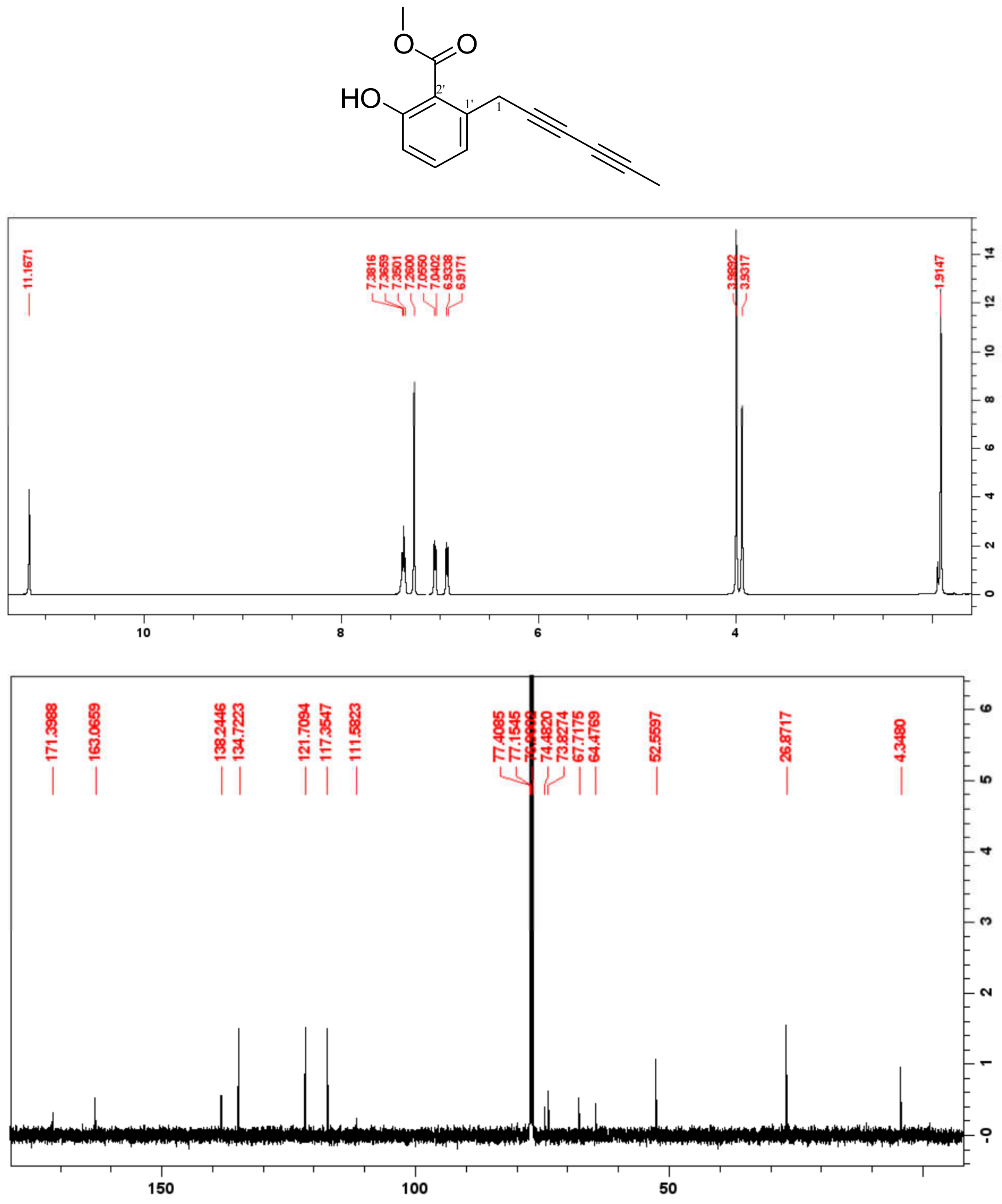

Figure S10. ^{1}H and ^{13}C NMR spectra of **10** (3′-*O*-demethyl-frutescin) in $CDCl_3$. (600 and 150 MHz, respectively).

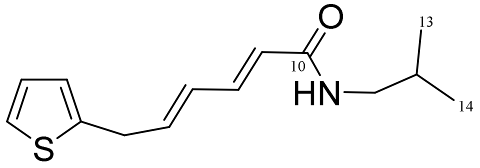

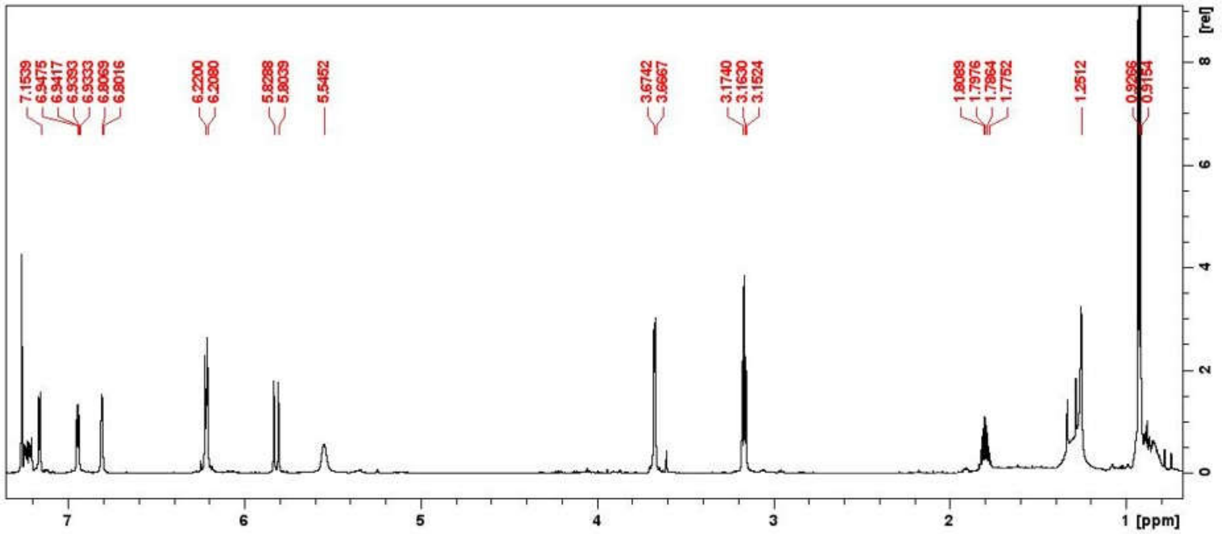

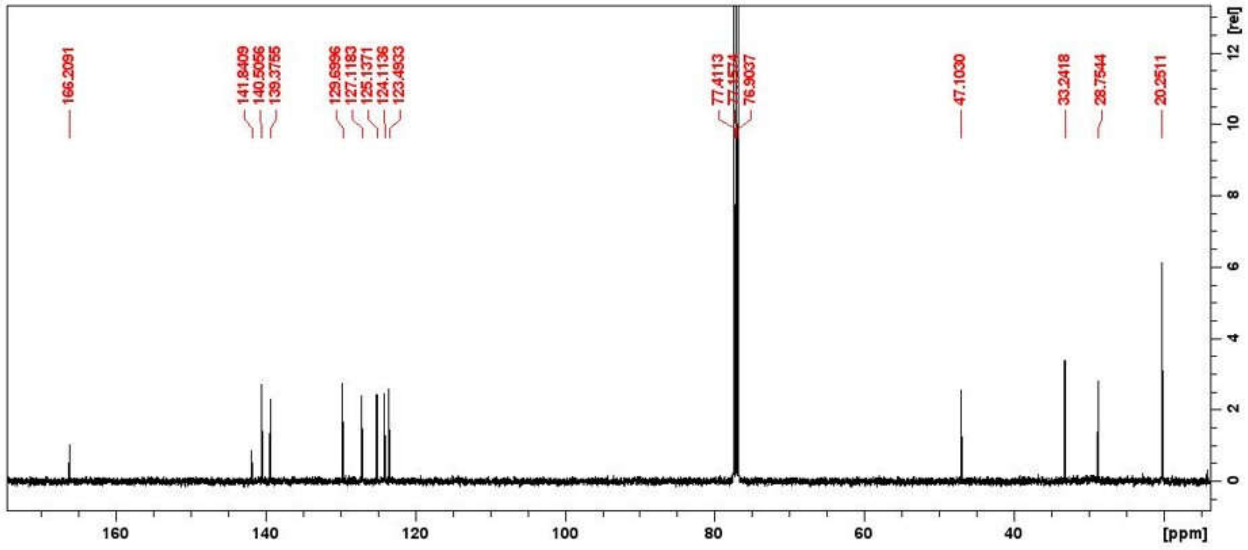

Figure S11. ^{1}H and ^{13}C NMR spectra of **11** [(*N*-isobutyl-6-(2-thienyl)-2E,4E-hexadienamide] in $CDCl_3$ (500 and 125 MHz, respectively).

(A) Before saturation with with barium (II) salt

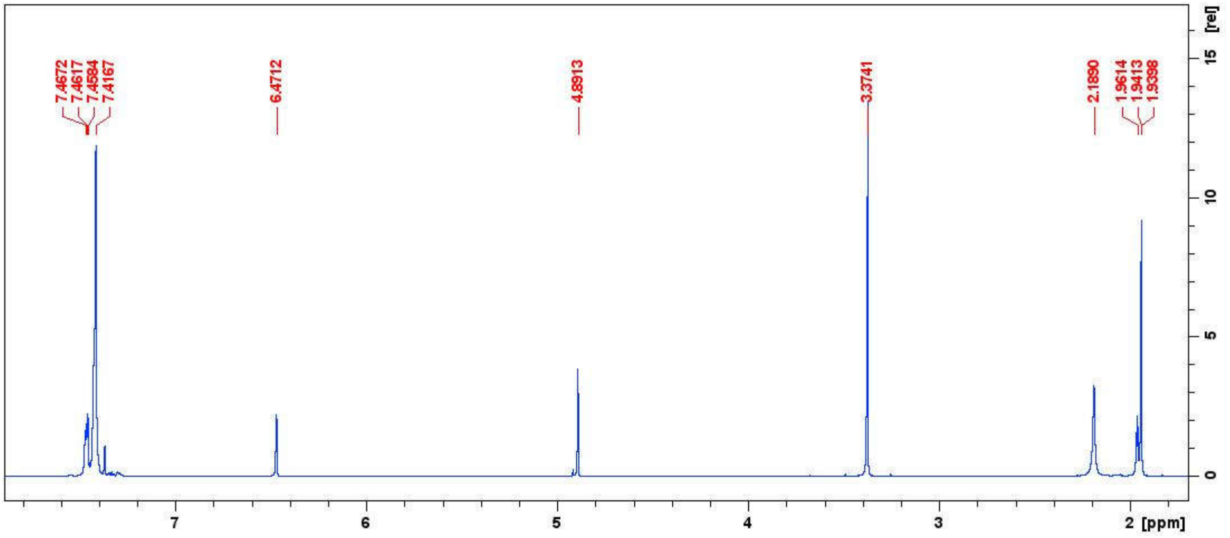

(B) After saturation with with barium (II) salt

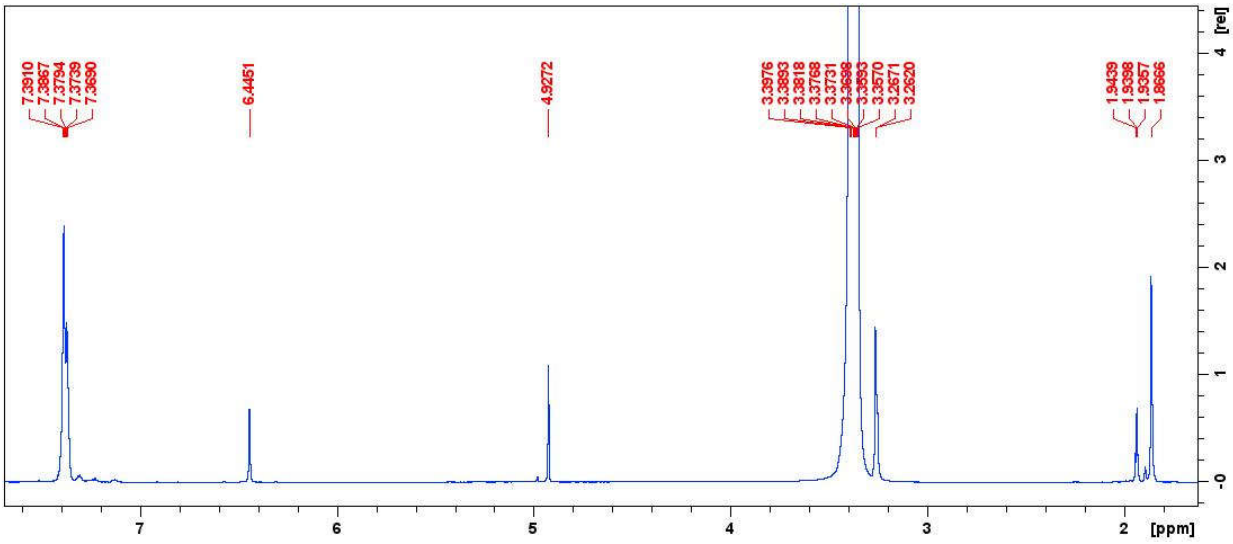

Figure S12 . ^{1}H NMR spectra of capillinol (*R*)-(-)-α-methoxyphenylacetate before (**A**) and after (**B**) saturation with barium (II) salt as the chelating agent.

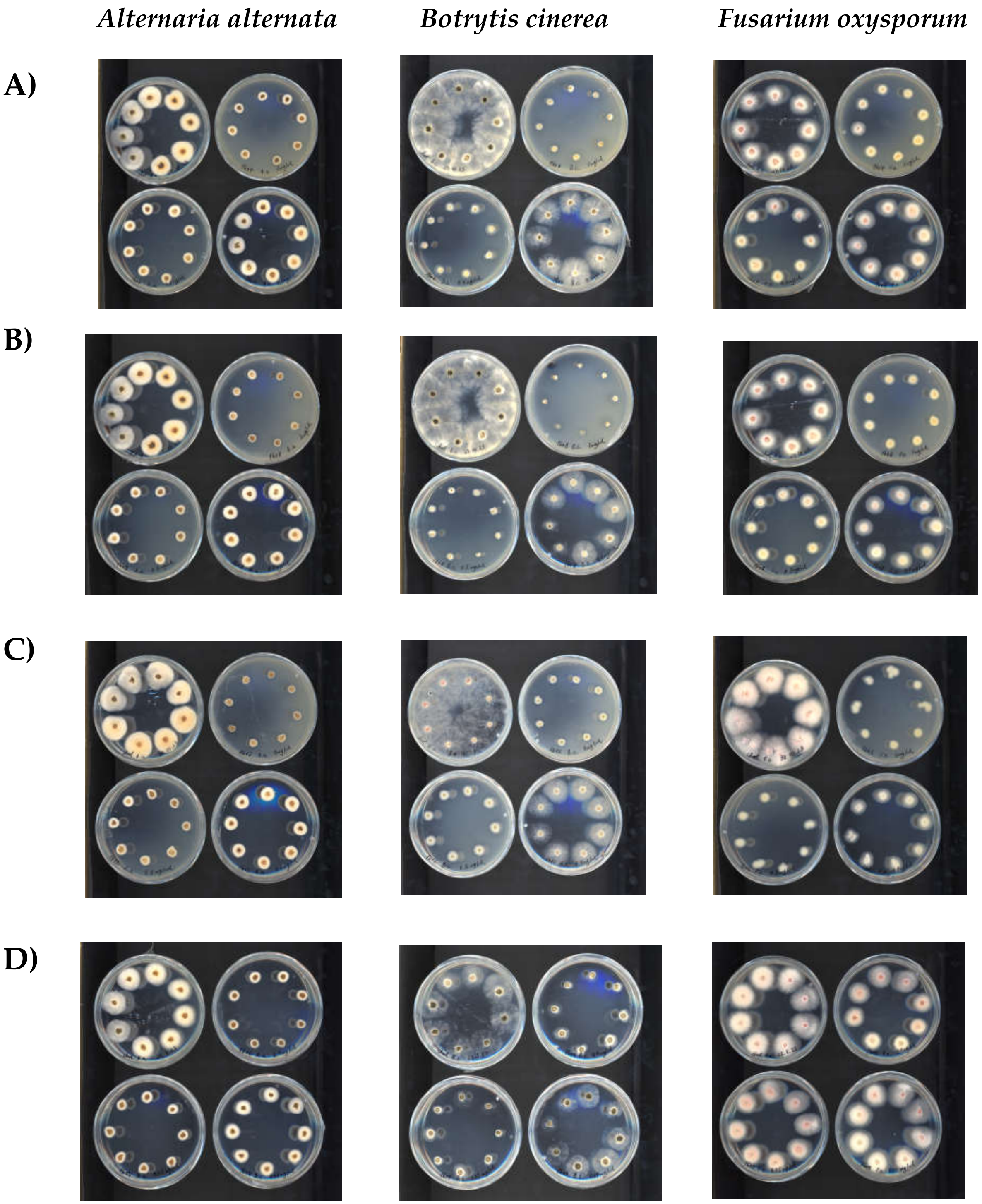

Figure S13. Representative photographs of fungal growth inhibition in a dilution agar assay using: **A)** Ethanolic extract, **B)** Hexane fraction and **C)** Subfraction B2 from cultivated *Argyranthemum frutescens* against *Alternaria alternata*, *Botrytis cinerea* and *Fusarium oxysporum* at 1, 0.5, and 0.1 mg/mL, and D) Compound **4** at 0.1, 0.05, and 0.01 mg/mL. In all examples, plate on the top left corresponds to the negative control, at the top right is the sample at the higher concentration, and at the bottom right is the lower assayed concentration.

Table S1. Antifungal effects (% Growth Inhibition) of extract, fractions, and sub-fractions from roots of wild *Argyranthemum frutescens* against *Alternaria alternata*, *Botrytis cinerea* and *Fusarium oxysporum.*

**Sample**	***A. alternata***			***B. cinerea***			***F. oxysporum***		
	1 mg/mL	0.5 mg/mL	0.1 mg/mL	1 mg/mL	0.5 mg/mL	0.1 mg/mL	1 mg/mL	0.5 mg/mL	0.1 mg/mL
Extract EtOH	55.7 ± 5.1	43.0 ± 3.7	NA	100.0 ± 0.0	86.3 ± 6.4	47.0 ± 3.4	90.2 ± 2.3	68.9 ± 5.6	16.0 ± 1.6
Fraction Hx	74.3 ± 5.6	69.8 ± 2.6	38.8 ± 2.6	100.0 ± 0.0	99.7 ± 0.3	94.3 ± 1.2	84.2 ± 5.6	77.0 ± 3.2	35.9 ± 2.6
A1	23.8 ± 2.3	21.1 ± 1.6	13.0 ± 0.8	44.0 ± 3.9	31.7 ± 2.5	7.2 ± 0.7	29.7 ± 2.9	17.6 ± 1.7	ND
A2	68.0 ± 1.8	54.2 ± 2.9	25.1 ± 2.5	100.0 ± 0.0	100 ± 0	68.5 ± 6.8	79.8 ± 3.5	66.2 ± 5.5	41.5 ± 3.8
A3	65.7 ± 1.1	55.1 ± 3.8	25.7 ± 2.4	100.0 ± 0.0	100 ± 0	72.0 ± 5.1	70.3 ± 2.7	63.9 ± 4.0	35.6 ± 3.4
A4	65.0 ± 3.3	65.1 ± 2.5	44.4 ± 3.4	100.0 ± 0.0	100 ± 0	98.7 ± 1.1	81.2 ± 3.4	80.7 ± 3.9	43.8 ± 3.4
A5	49.3 ± 4.0	43.7 ± 3.4	21.2 ± 2.0	92.0 ± 2.9	85.7 ± 5.8	29.9 ± 2.7	46.7 ± 2.5	33.1 ± 2.2	18.3 ± 1.8
A6	39.8 ± 1.0	38.8 ± 1.5	25.5 ± 1.1	81.3 ± 1.9	69.5 ± 2.7	43.0 ± 4.1	53.2 ± 2.0	41.6 ± 2.2	27.7 ± 2.3
A7	22.7 ± 1.8	17.7 ± 1.6	ND	50.7 ± 3.2	41.6 ± 4.2	34.4 ± 3.4	32.7 ± 1.8	30.7 ± 3.0	18.1 ± 1.2
A8	NA	ND	ND	25.2 ± 1.9	29.0 ± 2.5	28.7 ± 2.8	20.6 ± 2.0	19.2 ± 1.6	NA
Fraction EtOAc	53.1 ± 2.4	42.0 ± 4.15	15.8 ± 1.5	82.4 ± 3.0	77.9 ± 1.1	50.5 ± 4.8	57.6 ± 5.7	52.3 ± 4.7	37.4 ± 2.7
Fraction H_2O	NA	NA	NA	41.9 ± 3.9	NA	NA	NA	NA	NA
Fosbel-Plus	93.5 ± 3.6	87.8 ± 5.5	74.2 ± 4.8	83.3 ± 3.0	73.5 ± 5.8	23.6 ± 4.8	93.4 ± 4.8	92.6 ± 6.5	78.8 ± 4.7
Azoxystrobin	38.4 ± 3.7	33.4 ± 2.73	30.4 ± 2.1	75.1 ± 3.1	75.0 ± 3.0	67.8 ± 4.1	58.7 ± 4.6	56.1 ± 4.1	53.2 ± 5.1

Extract/fractions/subfractions with an inhibition growth higher than 20% at 1 mg/mL were assayed at lower concentrations (0.5 and 0.1 mg/mL). % Growth Inhibition: Means ± standard deviation (SD). NA: not active (% inhibition ≤10); ND: not determined. Fosbel-Plus and Azoxystrobin were used as positive controls.

Table S2. Antifungal effects (% Growth Inhibition) of extract, fractions, and sub-fractions from roots of cultivated *Argyranthemum frutescens* against *Alternaria alternata*, *Botrytis cinerea* and *Fusarium oxysporum*.

**Sample**	***A. alternata***			***B. cinerea***			***F. oxysporum***		
	1 mg/mL	0.5 mg/mL	0.1 mg/mL	1 mg/mL	0.5 mg/mL	0.1 mg/mL	1 mg/mL	0.5 mg/mL	0.1 mg/mL
Extract EtOH	67.9 ± 2.2	60.5 ± 3.2	39.6 ± 3.7	93.4 ± 1.7	82.9 ± 3.1	33.8 ± 3.4	58.6 ± 3.7	47.7 ± 2.3	14.9 ± 2.7
Fraction Hx	79.1 ± 2.9	66.5 ± 3.7	46.8 ± 2.5	99.6 ± 0.8	91.8 ± 3.5	46.1 ± 3.7	64.1 ± 2.3	52.3 ± 3.6	27.6 ± 3.0
B1	72.4 ± 4.6	65.9 ± 4.0	20.0 ± 2.5	99.8 ± 0.4	95.2 ± 2.4	46.3 ± 3.1	66.3 ± 2.0	60.4 ± 1.4	36.1 ± 3.0
B2	86.7 ± 1.9	74.7 ± 2.5	59.3 ± 2.8	90.1 ± 0.5	81.9 ± 1.1	42.4 ± 2.3	65.1 ± 1.0	62.6 ± 1.5	45.8 ± 3.9
B3	91.5 ± 8.9	70.0 ± 3.1	59.6 ± 4.1	96.2 ± 0.4	85.2 ± 3.0	48.7 ± 3.5	65.2 ± 2.2	57.5 ± 4.1	38.2 ± 3.5
B4	58.2 ± 4.0	53.8 ± 4.1	45.8 ± 3.7	87.1 ± 3.4	72.4 ± 3.4	32.4 ± 3.3	63.5 ± 1.7	41.3 ± 1,7	21.3 ± 1.7
B5	48.5 ± 4.4	28.5 ± 2.6	NA	89.0 ± 5.4	78.4 ± 5.8	75.4 ± 2.5	32.8 ± 3.6	32,2 ± 2.4	15.8 ± 1.4
B6	NA	NA	NA	34.3 ± 3.4	22.6 ± 2.2	NA	11.1 ± 1.1	10.2 ± 1.0	NA
B7	NA	NA	NA	40.9 ± 3.0	18.7 ± 1.8	16.2 ± 2.2	20.7 ± 1.4	19.4 ± 1.1	16.3 ± 2.2
Fraction EtOAc	58.8 ± 1.4	53.3 ± 3.4	32.1 ± 3.2	78.7 ± 5.5	61.1 ± 3.2	42.3 ± 4.1	53.8 ± 1.9	44.4 ± 4.1	18.7 ± 1.7
Fraction H_2O	NA	NA	NA	75.6 ± 4.3	16.9 ± 1.6	10.5 ± 1.0	29.6 ± 2.2	33.5 ± 2.6	20.2 ± 1.5
Fosbel-Plus	93.5 ± 3.6	87.8 ± 5.5	74.2 ± 4.8	83.3 ± 3.0	73.5 ± 5.8	23.6 ± 1.8	93.4 ± 4.8	92.6 ± 6.5	78.8 ± 4.7
Azoxystrobin	38.4 ± 3.7	33.4 ± 2.7	30.4 ± 2.1	75.1 ± 3.1	75.0 ± 3.0	67.8 ± 4.1	58.7 ± 4.6	56.1 ± 4.1	53.2 ± 5.1

Extract/fractions/subfractions with an inhibition growth higher than 20% at 1 mg/mL were assayed at lower concentrations (0.5 and 0.1 mg/mL). % Growth Inhibition: Means ± standard deviation (SD). NA: not active (% inhibition ≤10); ND: not determined. Fosbel-Plus and Azoxystrobin were used as positive controls.
